# Nuclear export of chimeric mRNAs depends on an lncRNA-triggered autoregulatory loop in blood malignancies

**DOI:** 10.1038/s41419-020-02795-1

**Published:** 2020-07-23

**Authors:** Zhen-Hua Chen, Tian-Qi Chen, Zhan-Cheng Zeng, Dan Wang, Cai Han, Yu-Meng Sun, Wei Huang, Lin-Yu Sun, Ke Fang, Yue-Qin Chen, Xue-Qun Luo, Wen-Tao Wang

**Affiliations:** 1 MOE Key Laboratory of Gene Function and Regulation, State Key Laboratory for Biocontrol, School of Life Sciences, Sun Yat-sen University, 510275 Guangzhou, China; 2 Sun Yat-sen University Cancer Center, State Key Laboratory of Oncology in South China, 510060 Guangzhou, Guangdong China; 3 The First Affiliated Hospital, Sun Yat-sen University, 510080 Guangzhou, China

**Keywords:** Acute myeloid leukaemia, RNA transport

## Abstract

Aberrant chromosomal translocations leading to tumorigenesis have been ascribed to the heterogeneously oncogenic functions. However, how fusion transcripts exporting remains to be declared. Here, we showed that the nuclear speckle-specific long noncoding RNA MALAT1 controls chimeric mRNA export processes and regulates myeloid progenitor cell differentiation in malignant hematopoiesis. We demonstrated that MALAT1 regulates chimeric mRNAs export in an m6A-dependent manner and thus controls hematopoietic cell differentiation. Specifically, reducing MALAT1 or m6A methyltransferases and the ‘reader’ YTHDC1 result in the universal retention of distinct oncogenic gene mRNAs in nucleus. Mechanically, MALAT1 hijacks both the chimeric mRNAs and fusion proteins in nuclear speckles during chromosomal translocations and mediates the colocalization of oncogenic fusion proteins with METTL14. MALAT1 and fusion protein complexes serve as a functional loading bridge for the interaction of chimeric mRNA and METTL14. This study demonstrated a universal mechanism of chimeric mRNA transport that involves lncRNA-fusion protein-m6A autoregulatory loop for controlling myeloid cell differentiation. Targeting the lncRNA-triggered autoregulatory loop to disrupt chimeric mRNA transport might represent a new common paradigm for treating blood malignancies.

## Introduction

Genomic alterations, particularly aberrant chromosomal translocations, are responsible for the onset of many types of cancers^1,2^, such as leukemia. For example, *PML-RARα* (t15; t17)^3,4^, *MLL* fusions (t11)^5–7^, and *AML1-ETO* (t8; t21)^8–10^ are typical oncogenic fusion genes that contribute to particular subtypes of leukemogenesis. How these rearrangements can lead to tumorigenesis has traditionally been explained by their ability to encode and express proteins; such proteins are commonly referred to as oncogenic ‘fusion proteins’^2,11^, indicating that the regulation of mRNA export of fusion proteins is important for oncogenic protein expression. However, how the resulting chimeric mRNA exports from the nucleus to the cytoplasm, as well as whether such alterations of the genome in the nucleus could also simultaneously impact the nuclear architecture, has been poorly characterized to date. Importantly, regulation of RNA exporting process has been shown to contribute to the drug-induced eradication of cancer cells^12^. Thus, a greater understanding of the mRNA export process of chromosomal translocations resulting in fusion genes is especially important for clinical therapeutic drug development^11,13^.

It has been generally considered that most of mRNA exports are modulated by TAP-p15 heterodimers^14–16^, as well as a series of mRNA-binding proteins and adaptor proteins^14,17^. Recent studies have shown that the mRNA export process is also characterized by modifications of pre-mRNA, including N6-methyladenosine (m6A) modifications^18–20^. The identified m6A methyltransferase complex comprising of methyltransferase-like 3 (METTL3), METTL14, and Wilms’ tumor 1-associating protein (WTAP) has been found to be specifically located in nuclear speckles^21^, a nuclear structure related to mRNA export^17,22–25^. In addition, nuclear noncoding RNAs (ncRNAs) are emerging as essential regulators of mRNA export^26–29^. The long noncoding RNA (lncRNA) NEAT1 is reportedly responsible for retaining double-stranded mRNAs in the nucleus^26–29^, suggesting that the mRNA export process is under complex regulatory control. Very interestingly, autoregulatory loops often provide precise control of the mRNA-exporting process of specific genes that encode key proteins^30,31^. For example, nuclear YRA1 autoregulates its own mRNA in trans and commits its pre-mRNA to the nuclear export process^32^. Fusion protein partners, such as PML and AF4, are reported to have the capacity to bind to nascent RNA and their own mRNAs in the nucleus^33–36^. The questions how fusion transcripts are exported from nucleus to cytoplasm and whether they are in potential autoregulation loop systems remain to be declared.

In this study, we performed genome-wide screening via RNA immunoprecipitation sequencing (RIP-seq) assay to assess fusion protein-associated RNAs in the nucleus. We discovered that MALAT1 directly interacted with several fusion proteins, including PML-RARA, MLL-AF9, MLL-ENL, and AML1-ETO in nuclear speckles and affected the chimeric mRNA nucleocytoplasmic export and their protein production. We further showed that MALAT1 acted as a modulator that promotes fusion protein and m6A methyltransferase interactions, which in turn control the chimeric mRNA-exporting process through the m6A reader YTHDC1. This study is the first to demonstrate that nuclear export of chimeric mRNAs depends on an lncRNA-triggered autoregulatory loop. The universal m6A-dependent autoregulatory mechanism of mRNA transport of distinct fusion proteins revealed in this study may provide a new therapeutic paradigm for treatments of these leukemia subtypes.

## Materials and methods

### Cell lines and cultures

Leukemia cells (NB4, Molm13, Kasumi-1, HL60, and K562) and HEK-293T cells were purchased from American Type Culture Collection (ATCC, USA.) and were cultured in RPMI medium modified (HyClone, USA) and DMEM (HyClone, USA), respectively, containing 10% fetal bovine serum (Thermo Fisher Scientific, USA). The cells were cultured in a humidified atmosphere containing 5% CO_2_ at 37 °C.

The primary leukemia sample was obtained from informed consent from the first Affiliated Hospital of Sun Yat-sen University and was approved by the Hospital’s Protection of Human Subjects Committee.

### FACS analysis

Cell differentiation was assessed by detecting the surface antigen expression of CD11b, CD14, and CD15 (eBioscience, USA) by flow cytometric analysis using a BD FACSAria cytometer (BD Biosciences, USA).

### RNA isolation and quantitative real-time PCR

Total RNA from leukemia cells was extracted using TRIzol reagent (Invitrogen, USA) according to the manufacturer’s guidelines. RNA separation of nucleus and cytoplasm is performed according to the previous report methods^37^. Real-time PCR was performed to quantify mRNA expression using SYBR^®^ Premix Ex TaqTM (TliRNaseH Plus) (Takara, Japan) according to the manufacturer’s instructions. The qRT-PCR was performed to detect different fusion protein transcripts, m6A methyltransferases mRNA and lncRNA MALAT1 expressions. Briefly, RNA was reverse-transcribed to cDNA using a PrimeScriptTM RT reagent kit with gDNA Eraser (Perfect Real Time) (Takara, Japan), and amplified with specific mRNA RT and PCR amplification primers. GAPDH served as internal controls. For the fusion proteins’ mRNAs, such as *PML-RARα, MLL-AF9, BCR-ABL1*, and *AML1-ETO*, primers were designed as previously reported. The oligonucleotide sequences are shown in Supplementary Table S1.

### Constructs

The human METTL3, METTL14, WTAP, and SRSF3 CDS were obtained in human cells and the primers were designed and synthesized according to the previous reports. PML-RARA full length was amplified based on the total RNA from the NB4 cells. MLL-ENL, AML1-ETO, BCR-ABL full length expression plasmid were purchased from addgene. All the primers used for plasmid constructions were showed in Supplementary Table S1. The lentiviral shRNA expression plasmids targeting MALAT1 was performed according to the manufacture’s protocol of pGreenPuro™ shRNA Cloning and Expression Lentivector (System Biosciences, SBI). The lentiviral overexpression plasmids of METTL3, METTL14, and WTAP were designed and carried out with the PCDH lentivirus plasmid from addgene. PML-RARA-GFP gene was generated by homologous recombination and transferred to pcDNA3.1 which was also purchased from addgene. YTHDC1 and SRSF3 full length of CDS were amplified from human NB4 cells. PCR products were obtained and then cloned into pEASY-T3 (TransGen Biotech, China) for further sequencing.

### Transient transfection

For the IF and IP assays of target proteins overexpression, HEK-293T cells were seeded in six-well sterile plastic culture plates or a 60 mm diameter dish at a density of 3 × 10^5^ cells and 1.5 × 10^6^ per well, respectively, with complete growth medium. The cells were transfected with plasmids using Lipofectamine 3000 (Invitrogen) according to the manufacturer’s protocol. Small interfering RNAs (siRNAs) against human MALAT1 transcripts according to previous research and the negative control RNA duplex (RiboBio, China) were purchased from Guangzhou Ribo-Bio Co., Ltd. The siMALAT1-1 sequences were designed and expressed as shRNAs: sh-MALAT11. The siRNAs against human mRNA export adaptors were designed according to published data shown in Supplementary Table S2. Cells were transfected using the Neon^®^ Transfection System (Invitrogen, USA) with 100 pmol of oligonucleotides in 10 μl reactions according to the manufacturer’s guidelines.

### Western blot

Protein extracts were boiled in RIPA buffer (P0013B, Beyotime) and separated in a sodium dodecyl sulfate polyacrylamide electrophoresis (SDS–PAGE) gel. The proteins were then transferred to a polyvinylidene fluoride membrane (Millipore, USA) and probed with the primary antibodies, which are shown in Supplementary Table S3. Finally, the second antibody used and the reactive bands were detected by ECL (Thermo Scientific Pierce) in a dark room.

### RNA FISH

Different fluorescence labeled RNA probe targeting PML and RARα mRNAs was synthesized by Ribo-Bio (Cy3-PML mRNA) and Sangon Biotech (FITC-RARα mRNA). The targeting sequence of RARA is: ‘GCAAATACACTACGAACAACAGCTCAGAACAACGTGTC’. RNA FISH was carried out according to guidelines of Fluorescent In Situ Hybridization Kit (Ribo-Bio, China).

### Co-immunoprecipitation and MS2 Trap assay

Co-immunoprecipitation assay was performed as the published articles.

MS2 Trap procedure was carried out as previously described^38^.

### Immunofluorescence

The experiments are performed as our previous described procedures. Thirty-nine EM images were obtained from thin sections using a JEM1400 electron microscope (JEOL, Akishima, Tokyo, Japan). The relative cytosolic areas in cross-sections of 10 cells were analyzed by Image-Pro Plus software. And fluorescence signals obtained using anti-FLAG (mouse) (A8592, Sigma), anti-FLAG (rabbit) (20543-1-AP, Proteintech), anti-HA (mouse) (H3663, Sigma), anti-HA (rabbit) (C29F4, Cellular signaling), anti-SC35 antibody (Ab11826, Abcam), and anti-SF2 antibody (sc-33652, Santa Cruz) were acquired and analyzed by Carl Zeiss LSM 880 microscope (Carl Zeiss, Germany). At least 10 cells from each group were analyzed.

### RNA immunoprecipitation and sequencing

RNA immunoprecipitation was performed using a Magna RIP RNA-Binding Protein Immunoprecipitation Kit (Millipore, USA) according to the manufacturer’s instructions. Enriched total RNA was then prepared for the further sequencing and analysis of lncRNA and mRNA in Annoroad Company.

### MeRIP assay

Total RNA was isolated and then mRNA was collected according to the GenElute mRNA miniprep kit (Sigma, Cat. No. MRN10). mRNA of the samples was further performed according to the protocol of Magna MeRIP™ m6A Kit Transcriptome-wide Profiling of N6-Methyladenosine (Catalog no. 17-10499).

### Animal models

Five-week-old NOD-SCID male mice were maintained under specific pathogen-free conditions in the Laboratory Animal Center of Sun Yat-sen University, and all experiments on animals were performed according to the institutional ethical guidelines for animal experiments. The human APL-ascites NOD-SCID mice model and tail vein injection model were used as previously described. The tumor xenografts were established by a single intraperitoneal transplantation of 5 × 10^6^ NB4 cells in 0.2 ml of PBS into NOD-SCID mice. The NOD-SCID mice were intravenously (tail vein) implanted with sh-RNA-established NB4 cells. Direct injection of 5 × 10^6^ shRNA-transformed NB4 cells into 150 μL of PBS was performed to establish intravenous (tail vein) leukemia. The xenografted mice were randomized into different groups and the animal studies are not blinding. For the control, 150 μL of PBS without cells was injected. Subsequently, the BM from xenograft mice were treated with a red blood cell lysis buffer (Biolegend, USA), and cells washed with PBS containing 2% FBS before cell cytometry. Flow cytometry for the GFP+% of transduced NB4 cells was performed on a C6 cytometer (BD, USA) and analyzed using FlowJo software. Three remaining mice were used to perform the survival assay.

### Statistical analysis

Data are expressed as the means ± SD of three independent experiments. One-way ANOVA was performed to compare multiple groups, and Tukey’s multiple comparison test was used to analyze multiple comparisons. Leukemia-free survival was analyzed using the Kaplan–Meier method with a log-rank test. Two-tailed tests were used for univariate comparisons. For univariate and multivariate analyses of prognostic factors, a Cox proportional hazard regression model was used. *p* < 0.05 was considered statistically significant.

## Results

### Both the mRNA and fusion proteins resulting from chromosomal rearranged genes were hijacked in nuclear speckles by MALAT1

**Fig. 1:**
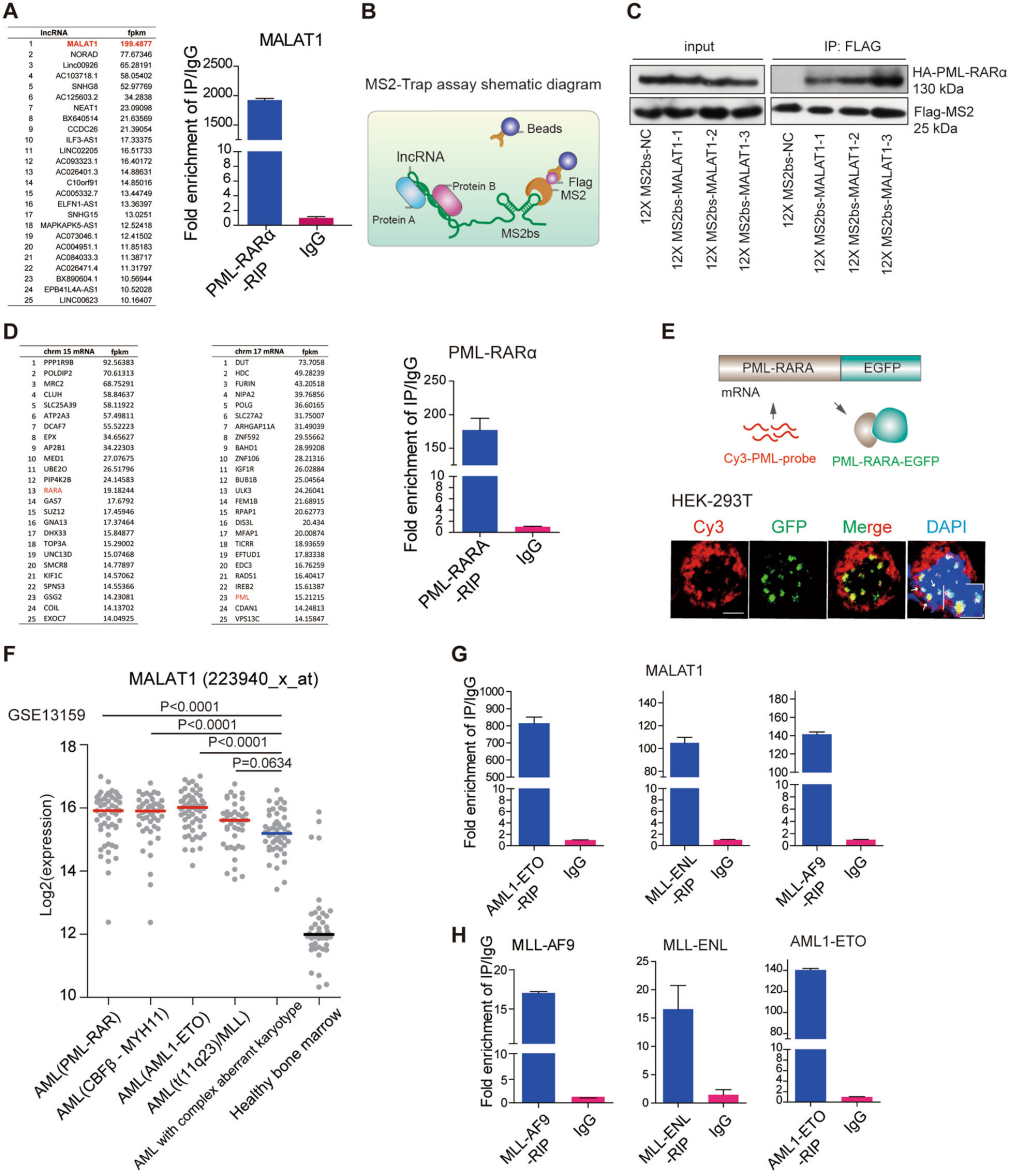
Both the mRNA and fusion proteins were hijacked in nuclear speckles by MALAT1. **a** The mRNAs and lncRNAs binding to PML-RARα (left panel). qRT-PCR validated MALAT1 binding to PML-RARA (right panel). **b** Schematic diagram showing the procedure of MS2-Trap assays. **c** PML-RARα interacted with MALAT1 in HEK-293T cells. The three sections of MALAT1 were named as MALAT1-1, MALAT1-2, MALAT1-3 respectively. **d** The mRNAs binding to PML-RARα. qRT-PCR confirmed the binding (right panel). **e** The mRNA FISH assay for detecting the interaction of PML-RARα and its own mRNA in HEK-293T cells in which PML-RARα-EGFP over-expressed. PML-RARα-EGFP (green) represents the fusion protein and PML-CY3 (red) represents mRNA transcripts. Scale bar represents 4 μm. **f** Reanalysis of the GES13159 data set with patient samples classified into many subtypes, including AML with t(11q23)/MLL, AML1-ETO, PML-RAR, and CBFb-MYH11. MALAT1 expression presented the highest levels in the AML patient subgroups with fusion proteins. **g**, **h** RIP assays followed by qRT-PCR analysis for the interaction of MALAT1 and the fusion protein mRNAs with fusion proteins (MLL-ENL, AML1-ETO, and MLL-AF9) in HEK-293T cells. Data are shown as the means ± s.e.m.; *n* = 3 independent experiments.

To determine if there are any regulatory RNAs or other molecules that fusion proteins directly interact with, we used the PML-RARα fusion protein as a model, which is a specific t(15;17) chromosomal translocation resulting in the fusion of the promyelocytic gene (PML) and the retinoic acid receptor alpha gene (RARA) into the oncoprotein PML-RARA^3,4^. We generated a stable NB4 cell line expressing FLAG-tagged PML-RARα using a lentivirus system in NB4 cells, and then performed RIP-seq assays to access the RNAs interacting with PML-RARα in cells. The specificity of the flag immunoprecipitation assay is shown in Supplementary Fig. S1a. RIP-seq identified a number of regulatory noncoding transcripts and mRNA potentially interacting with PML-RARα (Fig. 1a). Interestingly, MALAT1, a nuclear speckle-specific lncRNA, was identified as the most abundant ncRNA in deep sequencing data (Fig. 1a). To further validate this direct interaction and to determine the interacting residues of MALAT1, we carried out MS2-trap assay^38^ as shown in Fig. 1b using HA-tagged PML-RARα cDNA plasmid, which well expressed an endogenous fusion protein (Supplementary Fig. S1b); we also generated three partial MALAT1 constructs (Supplementary Fig. 1c). The results confirmed the interaction between MALAT1 and PML-RARα; specifically, the MALAT1 3′ end section interacted with PML-RARα much more tightly than the other sections (Fig. 1c), consistent with previous findings that MALAT1 functions primarily through its triple helix structure^39–41^.

In addition to binding to MALAT1, the RIP-seq data and qRT-PCR also showed that PML-RARα could bind to a number of mRNAs including their own mRNAs (Fig. 1d). In situ hybridization (FISH) assays further confirmed the direct interaction between PML-RARA and its own mRNA transcripts (Fig. 1e).

More remarkably, we found that MALAT1 displayed significantly higher expression levels in the AML patient subgroups with fusion proteins, including t(11q23)/MLL, AML1-ETO, PML-RAR, and CBFb-MYH11A, than those without (Fig. 1f), indicating that it may regulate the expression of chromosomal translocated genes in a common mechanism in the aggressive AML. Thus, to investigate the hypothesis, we further examined three other fusion proteins (MLL-ENL, MLL-AF9, and AML1-ETO). These translocations are typical oncogenic fusion genes that drive specific subtypes of leukemia^9,42,43^. The results further showed that MALAT1 and their own mRNAs were significantly enriched in the cells that overexpressed these three fusion proteins AML1-ETO, MLL-ENL, and MLL-AF9, respectively (Fig. 1g, h). These observations suggested that the MALAT1 interaction may be largely conserved in different cell types with different gene translocations. Because nuclear speckles are a subnuclear structure that is related to mRNA maturation processes, such as splicing and export, both the chimeric mRNAs and fusion proteins hijacked in nuclear speckles by MALAT1 raised the question whether MALAT1 is involved in the regulation of oncogenic fusion proteins or their mRNA maintenance or export?

### Nuclear export of chimeric mRNAs depends on the MALAT1 expression levels

**Fig. 2:**
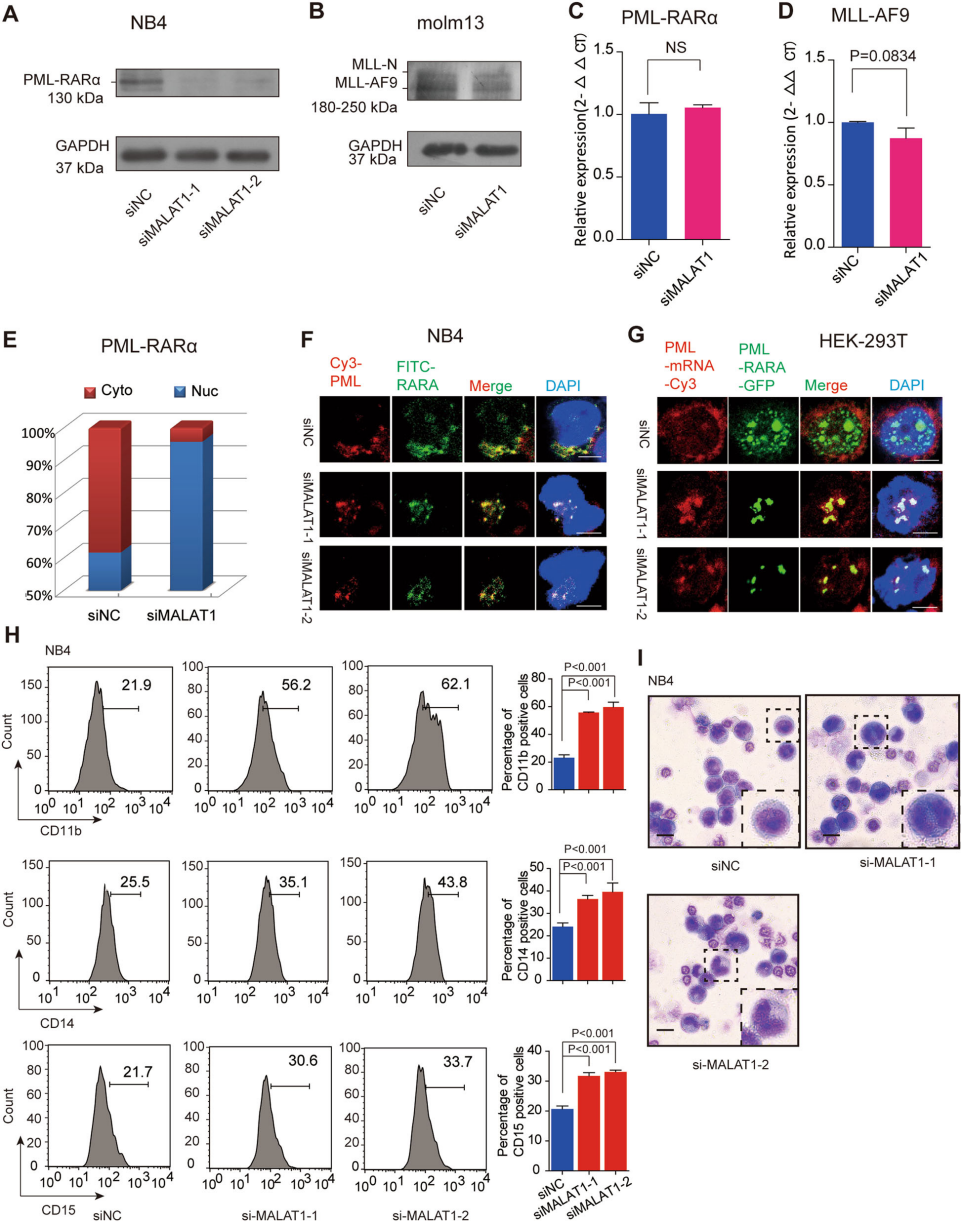
Knockdown of MALAT1 significantly repressed the expression levels of fusion proteins by promoting their mRNAs retention in nuclei. **a**, **b** Protein levels of PML-RARα and MLL-AF9 were reduced after knockdown of MALAT1 in NB4 cells and Molm13 cells, respectively. GAPDH was used as a loading reference. All experiments were performed independently at least three times. **c**, **d** qRT-PCR showed the mRNA levels of PML-RARα and MLL-AF9 were not markedly changed after reducing MALAT1 levels via siRNA. Data are shown as the means ± s.e.m.; *n* = 3 independent experiments. **e** PML-RARα mRNA detection by qRT-PCR after separating the nuclear and cytoplasmic RNAs. **f** PML-RARα mRNA location detecting by RNA FISH in NB4 cells. Two different fluorophores were added to PML (Cy3, red) and RARα (FITC, green) mRNA, respectively. The dots merged into yellow dots represent the mRNAs of PML-RARα. Scale bar represents 4 μm. **g** RNA FISH experiments were carried out according to the process shown in Fig. 1b in HEK-293T cells. Scale bar represents 4 μm. **h** Cellular differentiation markers CD11b, CD14, and CD15 were detected by flow cytometry. The results showed that compared to the negative controls, both markers were increased significantly when MALAT1 was down-regulated. The data were analyzed from at least three independent experiments and are shown as the means ± s.e.m. **i** Wright–Giemsa staining showed that cellular differentiation was significantly promoted after reducing MALAT1 levels in NB4 cells. Scale bar represents 20 μm.

To address whether MALAT1 regulates oncogenic fusion proteins or their mRNA export, we knocked down MALAT1 expression and then measured PML-RARα and MLL-AF9 protein levels, respectively. We found that both protein levels decreased sharply (Fig. 2a, b, and Supplementary Fig. S2a, b); however, the mRNA levels of PML-RARα and MLL-AF9 were almost identical before and after knocking down MALAT1 (Fig. 2c, d), raising the question that the decrease of fusion protein expression might not be related to the chimeric mRNA transcription, it might be related to the mRNA export and translation? To this end, nuclear and cytoplasmic RNAs were extracted and used for the chimeric mRNA detection. Notably, the mRNA of fusion protein PML-RARα was significantly retained in the nucleus when MALAT1 was reduced (Fig. 2e, and Supplementary Fig. S2c), while it has been rescued into cytoplasm when we overexpressed the MALAT1 3′ end section in MALAT1-silenced NB4 cells (Supplementary Fig. S2d, e). This observation was further confirmed by RNA FISH experiments as well (Fig. 2f). Moreover, in the cells that overexpressed an artificial PML-RARα-EGFP construct, knockdown of MALAT1 resulted in significant PML-RARα mRNA retention in nucleus (Fig. 2g), indicating that the binding of fusion proteins to their own mRNA was independent of MALAT1. These results support the hypothesis that MALAT1 promotes oncogenic fusion protein expression by facilitating chimeric mRNA export from nucleus to cytoplasm.

It has been well documented that oncogenic fusion proteins are key drivers of oncogenesis via the blockade of myeloid cell differentiation in myeloid leukemia^11,33,44^. Therefore, we investigated whether MALAT1 also plays a role in malignant progression. Knockdown of MALAT1 prominently facilitated the differentiation of AML cells to monocytes and granulocytes (Fig. 2h, and Supplementary Fig. S2f, g). Karyotype analysis by Wright–Giemsa staining showed that the nuclei appeared to be significantly lobulated when MALAT1 was suppressed (Fig. 2i, and Supplementary Fig. S2h, i) and apoptosis was significantly enhanced (Supplementary Fig. S2j). In summary, we concluded that MALAT1 maintained protein expression levels and oncogenic functions of fusion proteins by promoting their mRNA export from nucleus to cytoplasm.

### MALAT1 regulates chimeric mRNA export in a manner dependent on the YTHDC1 and SRSF3

**Fig. 3:**
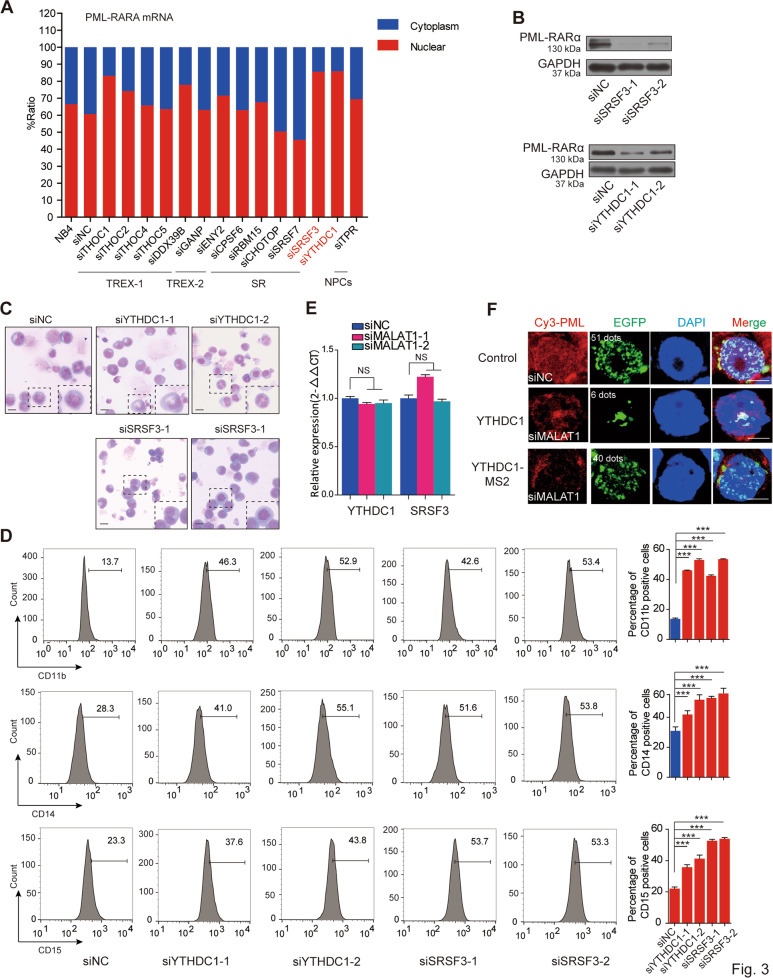
YTHDC1 and SRSF3 are responsible for the mRNA fusion gene export process. **a** Screening of mRNA export-related proteins that are specifically responsible for PML-RARα mRNA. **b** PML-RARα was diminished after knockdown of SRSF3 or YTHDC1 in NB4 cells. The data were obtained from three independent experiments. **c** Wright–Giemsa staining assays showed that phenotypes of the nuclei changed to a lobulated morphology after inhibiting YTHDC1 or SRSF33. Scale bar represents 20 μm. **d** Representative graphs for the flow cytometric analysis of myeloid markers of CD11b, CD14, and CD15 in NB4 when the YTHDC1 and SRSF3 were knocked down. Histogram plots show the statistical values. Error bars reflect ± SEM in three independent experiments. Error bars reflect ± SEM in three independent experiments (***p < 0.001). **e** YTHDC1 and SRSF3 transcripts expressions do not change significantly after knockdown of MALAT1 in NB4 cells. **f** Fusion mRNA transport process was dependent on YTHDC1. RNA FISH assay for testing PML-RARα mRNA transport by transfecting constructs of PML-RARα-EGFP-12×MS2bs and YTHDC1-MS2 upon knocking down MALAT1 in HEK-293T cells. Scale bar represents 4 μm. Data are shown as the means ± s.e.m.; *n* = 3 independent experiments.

We next asked how MALAT1 controls these chimeric mRNA export processes from nucleus to cytoplasm and regulates myeloid cell differentiation. Most mRNA export is specifically controlled by various protein factors and modifications of pre-mRNA, such as TAP-p15 heterodimers^15,16^, adaptor proteins^14^, and m6A modification^18,45^. Adaptor proteins include transcription-export-1 (TREX-1) complex^24,46,47^, TREX-2 complex^48^, and serine-arginine-rich (SR) proteins^49,50^. YTHDC1^51^, a ‘reader’ of m6A methylation, has been recently shown to regulate mRNA export processes by interacting with SR proteins^45^. Therefore, we chose 13 adaptor proteins (Supplementary Table S4) and used RNAi approaches to determine which adaptor is responsible for the chimeric mRNA export process in NB4 cells (the knockdown efficiency of these mRNAs have been shown in Supplementary Fig. S3a–c). We found that the amount of PML-RARα mRNA in nucleus was increased significantly in the adaptor knockdown cells compared with that in the control, such as SRSF3, YTHDC1, and THOC1 (Fig. 3a). Among the adapters, knocking down SRSF3 and YTHDC1 presented the most striking retention effect on PML-RARα mRNA in nucleus, suggesting that they could be the potential adaptor proteins responsible for PML-RARα mRNA export in NB4 cells. Furthermore, the protein level of PML-RARα was sharply decreased after that YTHDC1 or SRSF3 expression was reduced (Fig. 3b). We next performed Wright–Giemsa staining assays and flow cytometry to evaluate whether the YTHDC1 and SRSF3 complex is a key regulator in cellular functions (Fig. 3c). The results showed that cellular differentiation was markedly enhanced after knocking down YTHDC1 and SRSF3 (Fig. 3d), showing their regulatory roles in myeloid cell differentiation.

We next assessed the regulatory relationship between MALAT1 and YTHDC1 or SRSF3. No significant alterations of YTHDC1 and SRSF3 levels were found following reduction of MALAT1 levels (Fig. 3e). In addition, we designed a reporter vector to demonstrate whether the regulation of MALAT1 on the mRNA export process is dependent on the YTHDC1 recognition (Fig. 3f). The results showed that overexpression of YTHDC1 did not change the nuclear retention of PML-RARα mRNA after knockdown of MALAT1 (Fig. 3f). The results are similar with that of YTHDC1 when overexpressing SRSF3 under a MALAT1-silencing manner (Supplementary Fig. S3d, e). However, ectopic expression of MS2-tagged YTHDC1 could significantly enhance the export of PML-RARα mRNA (PML-RARα-EGFP-12×MS2bs), illustrating that YTHDC1 is crucial for the nuclear export of mRNA transcribed by fusion genes. These results suggest that MALAT1 might regulate chimeric mRNA export and hematopoietic differentiation in a manner dependent on the YTHDC1 and SRSF3 export-associated complex.

### YTHDC1 recognition of m6A modification on PML-RARα mRNA is required for its export

**Fig. 4:**
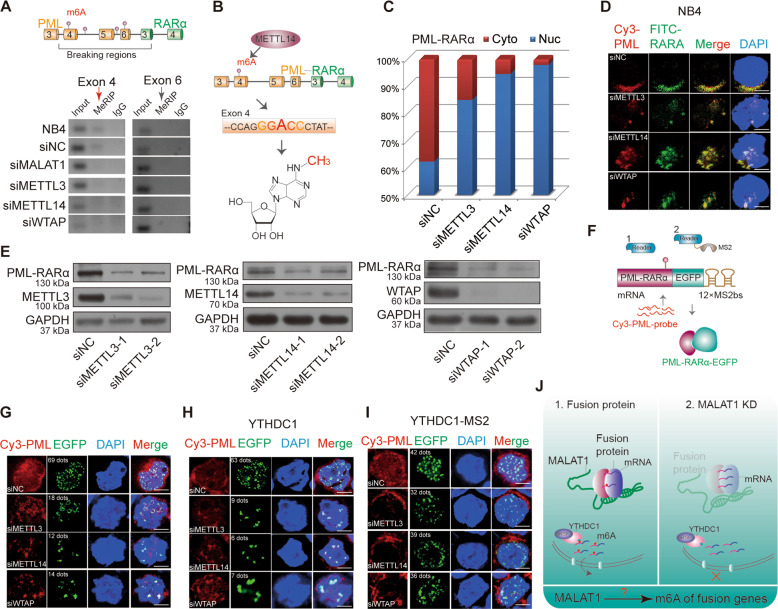
MALAT1-regulated m6A modification in the nucleus is responsible for the fusion gene export by YTHDC1. **a** A schematic showing potential m6A motifs in regions of PML-RARα mRNA. MeRIP assays were performed in NB4 cells after knockdown of m6A methyltransferases, MALAT1 or a negative control with siRNAs. Then, PCR sequencing for these sites, including potential motifs, were carried out; agarose electrophoresis was shown. **b** Schematic diagram of METTL14-methylated m6A sites in PML-RARα mRNA exon 4. **c** PML-RARA mRNA dispersion in nuclei and cytoplasm was detected after reducing the m6A level by knocking down the methyltransferases. **d** RNA FISH analysis showing the PML-RARα mRNA location in cells by two probes, Cy3-PML (red) and FITC-RARα (green). Scale bar represents 4 μm. **e** The protein levels of PML-RARα, METTL3, METTL14, and WTAP were monitored after knockdown of m6A methyltransferases by several different siRNAs. Three independent experiments were carried out. **f** Schematic diagram showing RNA FISH assays for testing PML-RARα mRNA transport by transfecting PML-RARα-EGFP-12×MS2bs and YTHDC1-MS2 constructs with knockdown of m6A in HEK-293T cells. **g** Representative images showed fusion protein green dots were reduced. Red dots were remained in nucleus when m6A methyltransferases were knocked down in HEK-293T cells. Scale bar represents 4 μm. **h** Combined with cotransfecting YTHDC1 into HEK-293T cells, representative images of PML-RARα mRNA and PML-RARα-EGFP are shown in HEK-293T cells after reducing m6A levels. Scale bar represents 4 μm. **i** Combined with cotransfecting YTHDC1-MS2, representative images showed PML-RARα mRNA retention in nuclei was significantly restored after reducing m6A, as compared with the negative controls. Scale bar represents 4 μm. **j** Schematic diagram showing the hypothesis the potential roles of MALAT1, m6A, and the reader YTHDC1 in the fusion gene export process.

YTHDC1, a member of the YTH domain-containing protein family, is reported to be an important m6A reader. Interestingly, a recent study showed that YTHDC1 facilitated m6A-methylated mRNA export by interacting with SRSF3; therefore, we proposed that the PML-RARα mRNA export process might be regulated by m6A modification. To evaluate this hypothesis, we first analyzed the sequence of PML-RARα mRNA to determine the conserved m6A motif. A few potential motifs for METTL14 and WTAP in the junction sites of PML-RARα (exons 4, 5, and 6 of the PML gene even including introns) were found (Fig. 4a, and Supplementary Table S5). Then, we performed the MeRIP assay with or without knockdown of MALAT1 and m6A methyltransferases, respectively. PCR results showed that the band representing sequences containing one of the motifs of METTL14 in PML exons 4, ‘GGACC’, was diminished following the reduction in m6A levels by knocking down methyltransferases (Fig. 4a and Supplementary Fig. S4a), indicating that the ‘A’ base in the motif was indeed modified by m6A methyltransferases (Supplementary Fig. S4b). Furthermore, the PML exon 4 m6A signal was diminished in the MALAT1-knockdown samples as well (Fig. 4a, b).

Consistently, as shown in Fig. 4c and Supplementary Fig. S4c, d, the diminished of m6A modification retained the PML-RARα mRNA in nucleus, and the total mRNA levels were unchanged. Similar results were obtained using RNA FISH experiments upon m6A methyltransferases or MALAT1 knockdown (Figs. 4d and 2f). Moreover, PML-RARα protein levels were reduced significantly after reducing the expression of m6A ‘writers’ (Fig. 4e, and Supplementary Fig. S4f). We have also found MLL-AF9 proteins, but not the mRNA levels, were downregulated when the m6A “writers” were knocked down (Supplementary Fig. S4e, f). M6A knockdown significantly increased nuclear PML-RARα mRNA levels in HEK-293T cells that overexpressed the PML-RARα construct (Fig. 4g). Furthermore, overexpressed YTHDC1 did not change the nuclear retention of PML-RARα mRNA after knockdown of m6A complex genes METTL3, METTL14 as well as WTAP (Fig. 4h), whereas forced expression of YTHDC1 (MS2-tagged) could significantly restore the levels of PML-RARα mRNA export; this finding indicated that YTHDC1 regulates nuclear export of mRNA of fusion genes in a m6A-dependent manner (Fig. 4i). Additionally, Wright–Giemsa staining assays showed that nuclei appeared to be significantly lobulated when m6A methyltransferases were suppressed (Supplementary Fig. S4g). Knocking down METTL3, METTL14, and WTAP can also promote AML cell differentiation (Supplementary Fig. S4h). In addition, overexpressed MALAT1 could significantly rescue cell differentiations that were triggered by the silencing of m6A methyltransferases in NB4 cells (Supplementary Fig. S5), showing their regulatory roles in myeloid cell differentiation.

In conclusion, m6A modification of PML-RARα mRNA, controlled by MALAT1, is a key signature for PML-RARα mRNA processing in nuclear speckles. MALAT1-regulated m6A modification, which is recognized by YTHDC1, is necessary for the export of PML-RARα and cellular functionality (Fig. 4j).

### MALAT1 and fusion protein complexes serve as a functional loading bridge for the interaction of chimeric mRNA and METTL14

**Fig. 5:**
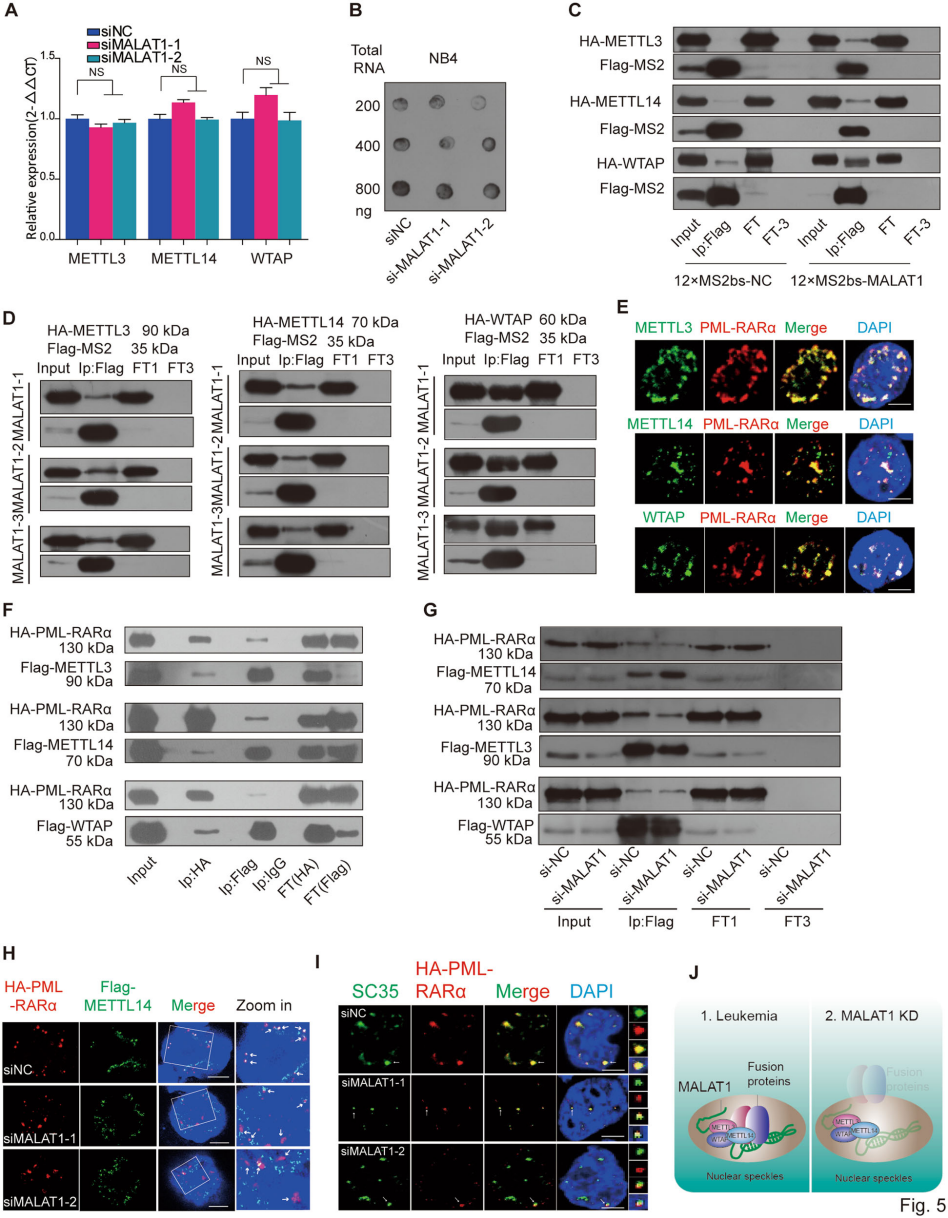
MALAT1 forms a complex with fusion proteins and m6A methyltransferases. **a** m6A methyltransferase expression was not regulated by MALAT1. Data are shown as the means ± s.e.m.; *n* = 3 independent experiments. **b** Dot blot assays were performed to determine cellular m6A levels after knockdown of MALAT1. **c** MALAT1 bound to m6A methyltransferases METTL3, 14 and WTAP directly by MS2-Trap assays in HEK-293T cells (FT1 flow through; FT3 flow through after three times wash).The experiments were performed independently at least three times. **d** MS2-Trap assays were carried out to test the interactions of METTL3, 14, and WTAP with the three sections of MALAT1. Flow through (FT1) and flow through after washing three times (FT3) were loaded as controls. **e** Immunofluorescence assays by laser confocal microscopy showed PML-RARα (anti-RARα) and m6A methyltransferases (anti-METTL3, 14, and WTAP) co-localized in NB4 cells. Scale bar represents 4 μm. **f** Co-immunoprecipitation and Western blot experiments were used to further detect the interaction of PML-RARα and m6A methyltransferases in HEK-293T cells (FT1 flow through; FT3 flow through after three times wash). **g** Co-immunoprecipitation of HA-tagged PML-RARA with Flag-tagged METTL3, 14, and WTAP in HEK-293T cells with MALAT1 knockdown by siRNA. Flow through (FT1) and flow through after washing three times (FT3) samples were loaded as controls. **h** HA-PML-RARα (anti-HA) and Flag-METTL14 (anti-Flag) localized in different positions in nuclei following knockdown of MALAT1 in HEK-293T cells by the methods of immunofluorescence. Scale bar represents 4 μm. **i** The nuclear localization of HA-PML-RARα (anti-HA) and nuclear speckle marker SC35 (anti-SC35) was only partially overlapping in the siMALAT1 samples compared to the negative controls in immunofluorescence assays. Representative images from confocal microscopy are shown from three independent experiments, scale bar represents 4 μm. **j** Schematic models indicating the potential mechanistic roles of fusion proteins and m6A methyltransferases in nuclear speckles.

The above results demonstrated that chimeric mRNA export is modulated by MALAT1 and m6A modification, which is recognized by YTHDC1. We next asked how MALAT1 regulates the m6A modification levels of fusion transcripts. Does MALAT1 affect the m6A methyltransferase expression levels? So we assessed m6A methyltransferase (METTL3, METTL14, and WTAP) levels following a reduction in MALAT1 expression in NB4 cells. However, the expression levels of the three m6A methyltransferases were not altered (Fig. 5a), and dot blot assays further supported this observation (Fig. 5b), indicating that m6A modification levels are not altered by MALAT1 directly. It has been previously reported that MALAT1 could bind to one of the m6A RNA methyltransferase members, WTAP^37^. Importantly, m6A methyltransferases are also located in nuclear speckles^21^. Thus, we hypothesized that MALAT1 might hijack fusion proteins in nuclear speckles to interact with the m6A modification complex and, in turn, regulate PML-RARα mRNA modification and export. Thus, we tested the interaction of m6A methyltransferases with MALAT1, as well as with fusion proteins. MS2-Trap assays showed that the m6A methyltransferases METTL3 and METTL14 could directly bind to three sections of MALAT1 (Fig. 5c, d). With immunofluorescent-labeling assays in NB4 cells and co-immunoprecipitation assays in HEK-293T cells, we found that PML-RARα and m6A methyltransferases also form a complex (Fig. 5e, f). We also constructed lentivirus-infected NB4 cells that stably express flag-tagged METTL3, METTL14, and WTAP to further validate this interplay. Supplementary Fig. S6 showed the binding of flag-tagged m6A methyltransferases and endogenous PML-RARα, which is consistent with that of antibodies for endogenous METTL3 and WTAP proteins.

Next, we investigated if MALAT1 mediates the interaction between PML-RARα and m6A complex components. The co-immunoprecipitation assays showed that the binding of METTL14 to PML-RARα was significantly reduced when MALAT1 level was decreased; however, the binding to METTL3 and WTAP was not significantly affected (Fig. 5g), indicating that METT14 is the main m6A methyltransferase component that might be of importance for PML-RARα mRNA modification, which is consistent with the data shown in Fig. 4b that METT14-modified motif sequence was found in PML-RARA mRNA. Immunofluorescence assays also showed that the overlapping areas of METTL14 and PML-RARα were reduced after treatment with siRNAs targeting MALAT1 (Fig. 5h). Very interestingly, when MALAT1 expression levels were reduced, PML-RARα dispersed near the nuclear speckles as a partially localized structure in HEK-293T cells with ectopic expression of PML-RARα (Fig. 5i), suggesting that MALAT1 might maintain their localization in nuclear speckles and their interaction with METTL14 (Fig. 5j). Therefore, MALAT1 and fusion protein complexes may serve as a functional loading bridge for the interaction of PML-RARα mRNA and m6A methyltransferases, further regulating modification and PML-RARα mRNA export.

### MALAT1 knockdown reduced fusion protein expression levels in primary cells and attenuated AML progression in vivo

**Fig. 6:**
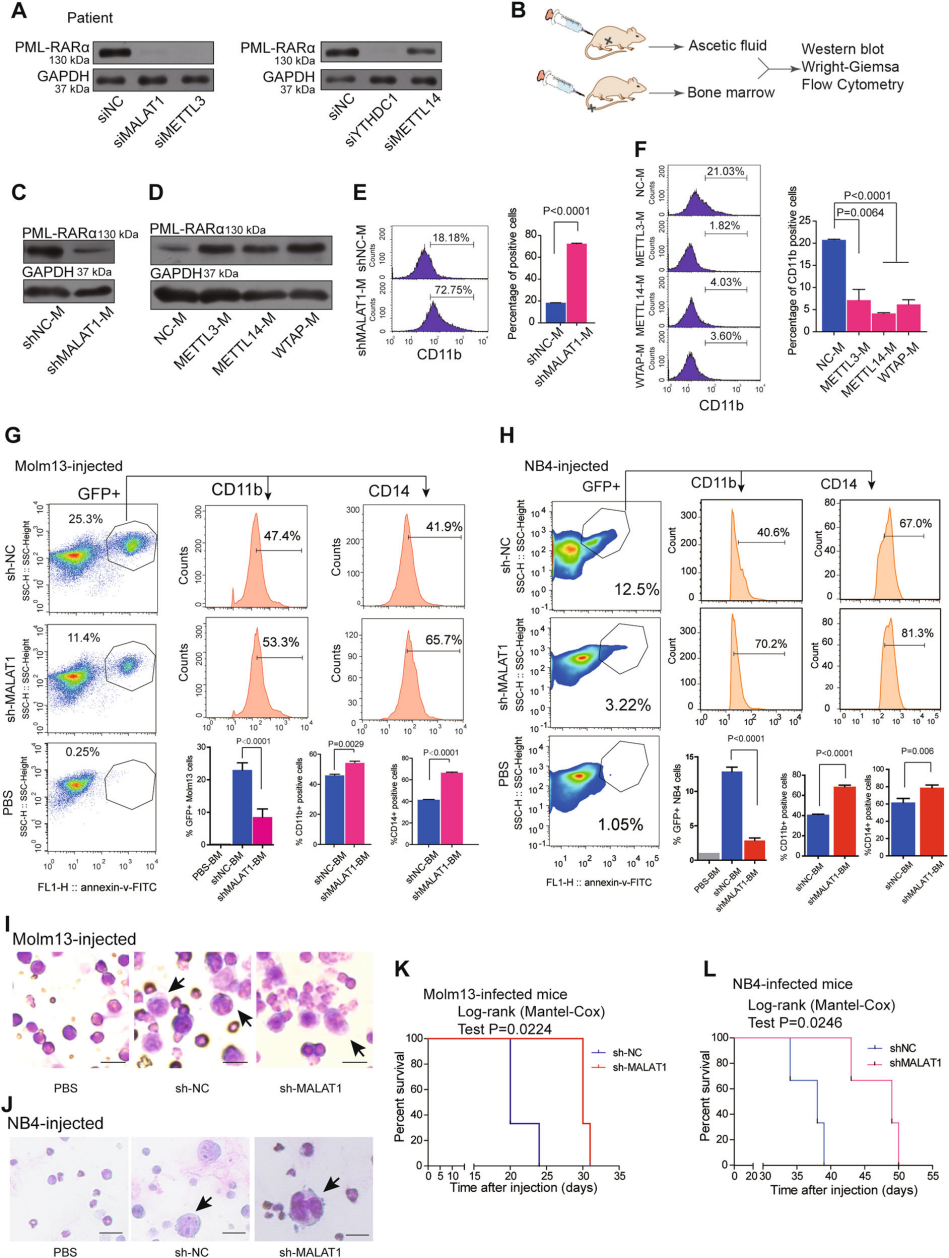
MALAT1 functions in cellular differentiation via m6A during malignant hematopoiesis in vivo. **a** The protein levels of PML-RARA in clinical patient #1 were determined via Western blot after knocking down MALAT1, m6A methyltransferases, and YTHDC1 with siRNAs. **b** Schematic diagram showing NOD-SCID murine models of ascites fluid and tail vein injection strategy, followed by Western blot, Wright–Giemsa, and flow cytometry analysis. **c**, **d** Western blot measurements of PML-RARA protein levels in the ascites fluid of mice injected with NB4 cells with suppressed MALAT1 (shMALAT1-M) or overexpressed m6A methyltransferases (METTL3-M, METTL14-M, WTAP-M). Three independent experiments were performed. **e**, **f** The differentiation marker CD11b of NOD-SCID murine ascites fluid was analyzed by flow cytometry after altering MALAT1 expression or m6A levels. Data are shown as the means ± s.e.m.; *n* = 3 independent experiments. **g**, **h** Differentiation marker CD11b and CD14 of BM cells of NOD-SCID mice intravenously injected by MLL-AF9-positive Molm13 cells were analyzed by flow cytometery after reducing MALAT1. Three mice each group, the mice were injected with the PBS as the negative control (*n* = 1). Data are analyzed and shown as the mean ± s.e.m.; *n* = 3 independent experiments. **i**, **j** Wright–Giemsa assays were carried out to verify that human Molm13 and NB4 cells (shNC and shMALAT1), which were much larger than mouse bone marrow cells, were transplanted to bone marrow. Scale bar represents 20 μm. **k**, **l** Survival curve of mice after intravenous injection of Molm13 and NB4 cells. Data shows that the survival time of mice injected with NB4 and Molm13 shMALAT1 cells significantly was longer than that in the control NB4 and Molm13 shNC cells. Three mice each group.

Finally, we collected primary cells from one clinical patient with PML-RARα for further functional validation. Upon knockdown of either MALAT1 or METTL3, the levels of PML-RARα were decreased significantly (Fig. 6a). We further established an ascites NOD-SCID mouse model using human leukemia cell line NB4 harboring PML-RARα to reveal the function of MALAT1 and m6A in vivo (Fig. 6b). Ascites NOD-SCID mice were produced 20 days after injecting 5 × 10^6^ lentivirus-infected NB4 cells with specific knockdown of MALAT1 (named NB4-Lv-NC or NB4-Lv-shMALAT1) or 5 × 10^6^ lentivirus-infected NB4 cells that overexpressed m6A methyltransferases (METTL3, METTL14, and WTAP), respectively. The stable knockdown and upregulation efficiencies of NB4-Lv-shMALAT1 and NB4-Lv-METT3, METT14, and WATP are shown in Supplementary Figs. S6 and S7a, b, respectively. The ascites fluid was collected and used for western blot and FACS analysis and Wright–Giemsa staining. Western blot analysis showed that PML-RARα was reduced when MALAT1 was knocked down in ascites fluid of mice (shMALAT1-M) (Fig. 6c and Supplementary Fig. S7c), whereas its level was markedly increased in the ascites fluid of m6A-enhanced NOD-SCID mice compared to that of controls (Fig. 6d). The nuclei were found to be lobulated and differentiated by Wright–Giemsa staining (Supplementary Fig. S7d, e). FACS analysis showed that CD11b and CD14 expression level were significantly induced (Fig. 6e, f, and Supplementary Fig. S7f). To further determine the effects of MALAT1 on PML-RARα in vivo, we also injected a NOD-SCID xenograft mice through tail vein with NB4 cells and moml13 cells that harboring MALAT1 short hairpin RNA (shMALAT1) or control short hairpin RNA (shNC). The mice were sacrificed after 21 days and the percentages of GFP^+^ cells were decreased in the bone marrow (BM) of mice treated with either the shMALAT1 NB4 cells or the shMALAT1 Molm13 cells compared with those in control mice (Fig.6g, h, left). In line with the CD11b expressions measured by flow-cytometry, reducing the level of MALAT1 significantly promote cell differentiation in the murine model, and another marker CD14 for differentiated cells confirms this phenomenon as well (Fig. 6g, h, right). Consistent with the pro-differentiation phenotype in vitro, impaired expression levels of the fusion protein PML-RARα were observed in BM cells of NB4-Lv-shMALAT1 injected mice rather than fusion protein levels in the BM cells of control mice (Supplementary Fig. S7g) Wright–Giemsa staining also verified that human leukemic cell, which are larger than bone marrow cells of mice, were induced to differentiate upon knocking down MALAT1 (Fig. 6i, j). Notably, the sh-MALAT1groups survived longer than the control groups (Fig. 6k, l), suggesting that MALAT1 knockdown could attenuate AML progression.

**Fig. 7:**
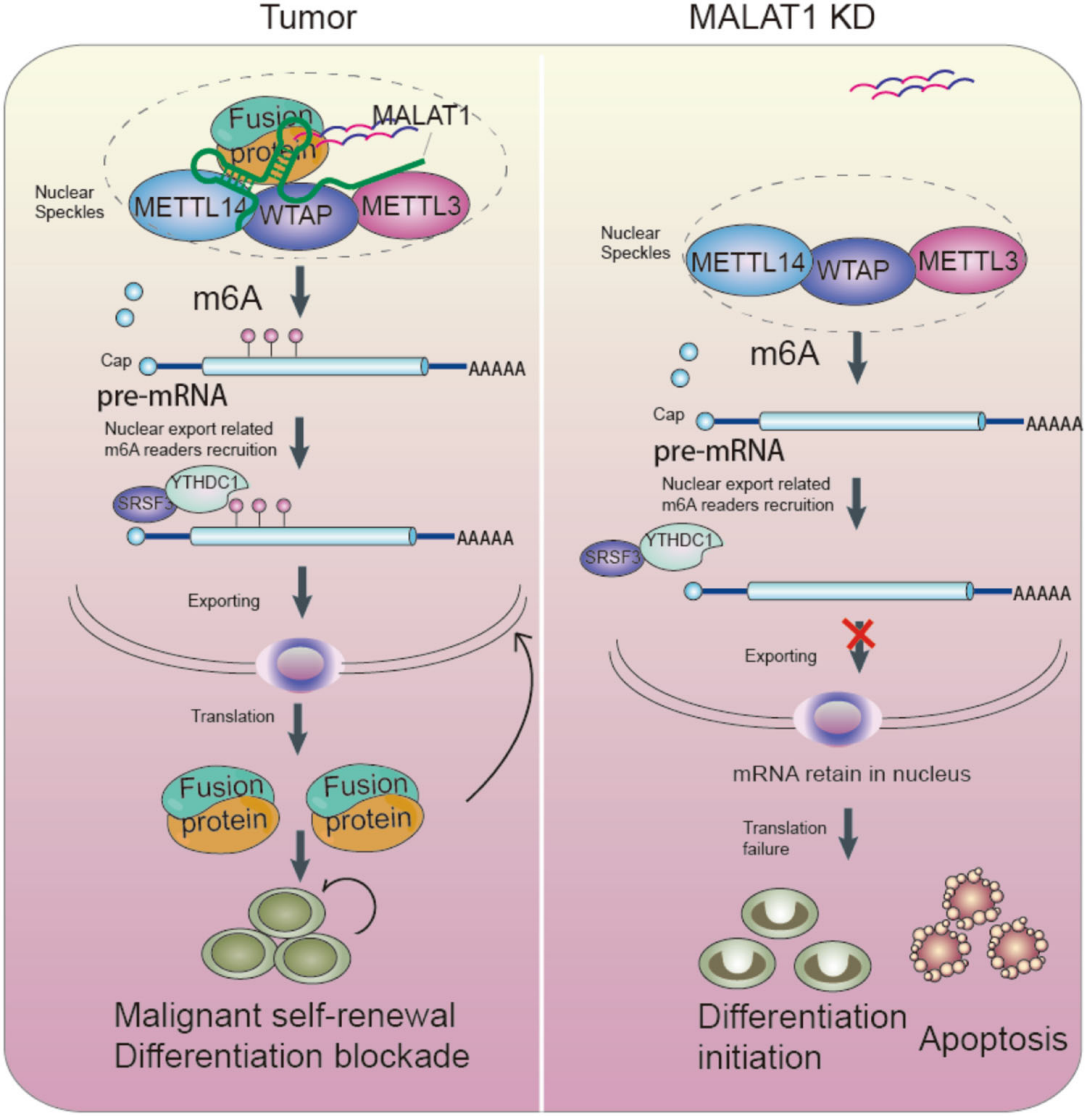
Schematic models for MALAT1 knockdown obstructed chimeric mRNA exporting and inhibit AML cell survival. Schematic models showing the mechanism of MALAT1-mediated regulation of the fusion gene export process by modulating the interaction of fusion proteins with METTL3, 14, and WTAP during hematopoiesis.

Collectively, we established that a functional RNP complex, which is composed of MALAT1, leukemia-related fusion proteins, and m6A methyltransferases, regulates the mRNA transcript export process in nuclear speckles. We revealed a lncRNA-fusion protein-m6A autoregulatory loop controls the chimeric mRNA export in malignant hematopoiesis (Fig. 7).

## Discussion

Fusion proteins resulting from aberrant chromosomal translocations have been determined to be key drivers for tumorigenesis^1,2,6^. In this study, it is the first to report that these fusion proteins were found to be located in nuclear speckles and physically interact with m6A methyltransferases (e.g., METTL3, METTL14, and WTAP) through the lncRNA MALAT1, an important regulatory component of the RNA–protein complex in nuclear speckles. We further discovered that the chimeric mRNA export was regulated by MALAT1 via m6A modification on their transcripts. Silencing MALAT1 could affect the export of these chimeric mRNAs from nucleus to cytoplasm, inhibiting the translation of fusion genes and ultimately controlling cell fates. The findings in this study showed that MALAT1 may be a potential therapeutic target for the treatment of fusion protein-driven cancers, and validation in additional clinical patients is needed in further studies. Furthermore, the study is the first to show lncRNA regulation of the chimeric mRNA export process.

LncRNAs, a subclass of ncRNAs with a sequence length >200 nt, have been shown to have multiple functions in various cellular processes through binding to chromatin-regulatory proteins^40,48^, DNA and even RNA on the basis of their secondary structures and/or primary sequences^9^. LncRNAs are also responsible for retaining of double-stranded mRNAs in nucleus^18,26–28^. For instance, NEAT1, a constitutive lncRNA in the nuclear paraspeckle, is responsible for the nuclear retention of inverted-repeat Alu element (IRAlu, double-stranded RNA structure) mRNAs by interacting with the A-I editing protein P54NRB. In contrast to the previously identified functions in the regulation of the alternative splicing process in nuclear speckles, MALAT1 is shown here to play a key role in the mRNA export process for the first time. We found that MALAT1 regulates typical chimeric mRNA export and determines lineage differentiation by directly binding to a complex of RNA modification-related proteins responsible for retaining double-stranded mRNAs in the nucleus. However, we have also found that knockdown of MALAT1 could facilitate the differentiation of HL60, an AML cell line without chromosomal translocation, implying that MALAT1 may also regulate the differentiation of myeloid cell without chimeric mRNA, independent of m6A pathways. The results further supported the multiple functions and regulatory mechanisms of MALAT1 in different cancers^52,53^.

Previous studies have shown that m6A modifications on the lncRNA XIST promote XIST-mediated X chromosomal transcriptional repression by interacting with the RNA-binding proteins RBM15 and RBM15B^54^, as well as the m6A methyltransferase WTAP^37^. Here, we found that MALAT1 induces specific m6A modification on the mRNAs by binding to these specific fusion proteins and m6A methyltransferases. Interestingly, it has been reported that a m6A modification at the MALAT1-2577 site, functioning as an RNA structural switch, regulates RNA–protein interactions^39,40^. Whether this m6A site contributes to the MALAT1-induced RNA and protein complex formation and function needs to be further investigated.

Finally, the study revealed the mechanism of the mRNA export process of chimeric mRNA includes lncRNA interactions with oncogenic fusion proteins, which in return autoregulate the m6A levels of their own mRNAs. The accurate intracellular interaction of proteins and their own mRNA has recently become regarded as a pervasive yet significant molecular mechanism^30,31^. More and more studies have shown that this interaction is a functional and regulatory model. Similarly, our work here revealed that the fusion protein localization and interaction with other regulatory proteins in nuclear speckles enables their own mRNA export from the nucleus to the cytoplasm. Furthermore, we found the lncRNA MALAT1 determined the interaction with RNA methyltransferases, resulting in the control of m6A levels in chimeric mRNAs that bound to proteins. Similar to the regulation of protein interactions by lncRNAs, overexpression of another ncRNA, e.g., pre-microRNA, resulted in nuclear mRNA retention and subsequent protein reduction of human Dicer levels (required for miRNA maturation) via competitive binding to Exportin-5 with Dicer mRNA^55^. In this study, we revealed that fusion proteins interact with RNA methyltransferases and subsequently autoregulate the export of their own mRNAs, binding to them via m6A. It could be concluded that, in the malignant hematopoiesis process, translocated proteins appear to form a positive feedback loop to maintain their persistent and ectopic expression by binding to their own mRNAs and promoting transport epigenetically. This study revealed that the novel lncRNA–fusion protein–m6A autoregulatory loop systems contribute to the chimeric mRNA export process and their eventual malignant functions.

In summary, this study showed that the nuclear lncRNA MALAT1 regulates the mRNA transport process from the nucleus to the cytoplasm via m6A modifications. MALAT1 promotes PML-RARA, MLL-ENL, MLL-AF9 and other fusion proteins to function in a dimensional RNA layer and further regulate the mRNA export process via the potential utility of chemical modifications of the mRNA. MALAT1, acting as a platform, modulates different translocations, resulting in oncogenic protein colocalization with m6A methyltransferases in nuclear speckles. YTHDC1, an m6A reader, regulates these chimeric mRNA export processes and functions in fusion protein-driven differentiation blockade and apoptosis inhibition. Targeting MALAT1 or the m6A reader YTHDC1 could retain oncogenic gene mRNAs in nuclei and contribute to the subsequent elimination of abnormally mutated malignant cells. The pathway described here is critical for leukemia and reveals a novel mechanism of lncRNA-mediated m6A modification and mRNA export of chimeric mRNA, shedding light on the multifunctional regulatory roles of lncRNAs in carcinogenesis. Therefore, targeting MALAT1 will be a promising therapeutic target for inhibiting fusion protein-driven cancer progression.

## Supplementary information

Supplementary tables

Supplementary Figure Legends

Figure S1

Figure S2

Figure S3

Figure S4

Figure S5

Figure S6

Figure S7
