# Supplementary material for: Nuclear export of chimeric mRNAs depends on an lncRNA-triggered autoregulatory loop in blood malignancies
Source: Cell Death Dis. 2020 Jul 23;11(7):566. doi: 10.1038/s41419-020-02795-1 (PMC7378249; doi:10.1038/s41419-020-02795-1)
Supplement: Supplementary file 1 — Supplementary tables [file 41419_2020_2795_MOESM1_ESM.docx]

**Supplementary Tables and legends**

**Supplementary Table S1. Primers used in qRT-PCR (QP) and full-length (FL) cDNA amplification.**

| Genes | Forward primer | Reverse primer |
| --- | --- | --- |
| MALAT1-1 | GAATTGCGTCATTTAAAGCCTAGTT | CCACTTCAAACTTCTATCTTCTCCA |
| MALAT1-2 | GGGAAAGCGAGTGGTTGGTA | AATCGGCCTACGTCCCCATA |
| MALAT1-3 | AGCAGACACACGTATGCGAA | GTGGTTCCCAATCCCCACAT |
| MALAT1-1-FL | TCTTAAGCGCAGCGCCATTTTAGC | CCAACTTCCCCTTCTAGCTTCAAT |
| MALAT1-2-FL | CTGGTGGTGCAGAAGTTAGAAG | AAGCCCACAGGAACAAGTCCTAC |
| MALAT1-3-FL | CATGTTAGGGATAAGTGCTTATTT | CAGTAGGGCTTCTCAAAACACC |
| GAPDH | GAGTCAACGGATTTGGTCGTAT | ATGGGTGGAATCATATTGGAAC |
| METTL3-QP | CAAGCTGCACTTCAGACGAA | GCTTGGCGTGTGGTCTTT |
| METTL14-QP | AGAAACTTGCAGGGCTTCCT | TCTTCTTCATATGGCAAATTTTCTT |
| WTAP-QP | TTCCCAAGAAGGTTCGATTG | TGCAGACTCCTGCTGTTGTT |
| YTHDC1-QP | TCTGGTTCAGGCACAGATGG | GGACAGCACGAACGGAAGAT |
| THOC1-QP | GCCCAAGTTTTGTGAAAGAAAGAAC | AGGGTCTGCCTGTTCAATGG |
| THOC2-QP | GCCAGTCGATACGGAAGGTT | ACTGCCCAGAGTAGCCCATA |
| THOC4-QP | AGGCCAAAACAACTTCCCGA | CTGTTCCTAAGCTGCGACCA |
| THOC5-QP | AGCCTCTTGATTGTGGAGGA | AGCTCTGGATCCTCCGTTCT |
| DDX39B-QP | GCTCTCCCTGTTTAGTTATGGC | GCACAGGACATCCATTCCCA |
| GANP-QP | TGAGCGAACCGGAAGTCGG | ACTCCAGAAGACGCTGGAAAG |
| ENY2-QP | AAGGGTCATTTCGTCGCTGG | TGGCATGCTGAGCAAGGAAT |
| CPSF6-QP | CGCATGTGAACCCAGCTTTC | CCAGAACCATAAGACTTGGACTCA |
| RBM15-QP | GCCTTCCCACCTTGTGAGTT | CTATAACAGGGTCAGCGCCAA |
| CHTOP-QP | CGGAATCGGTGGTAGAGGTC | GAGGATGGGCAGGCTTCAAT |
| SRSF7-QP | AGTCCAGATCTCCATCTCCAAA | AGATCCTGGCCAAGTTTTATGC |
| SRSF3-QP | AATTGGAACGGGCTTTTGGC | ATCGGGACGGCTTGTGATTTC |
| TPR-QP | CGTCTCCTAACCTGTGTCGT | AGTTGTTTTCCACCTTTTCTGAGG |
| C/EBPβ-QP | AACTCTCTGCTTCTCCCTCTG | AAGCCCGTAGGAACATCTTT |
| PU.1-QP | AGAAGAAGATCCGCCTGTACCA | GTGCTTGGACGAGAACTGGAA |
| NEAT1-QP | GTGGCTGTTGGAGTCGGTAT | TAACAAACCACGGTCCATGA |
| hY1-QP | GGCTGGTCCGAAGGTAGTGA | GCAGTAGTGAGAAGGGGGGA |
| PML-RARA-QP | ACCTGGATGGACCGCCTAG | GCTTGTAGATGCGGGGTAGAG |
| PML-exon 4-QP | TGCAGCTGTATCCAAGAAAGCC | CAGGTCAACGTCAATAGGGTCC |
| PML-exon 6-QP | CACCTCCAAGGCAGTCTCAC | TCACTTCCTATGACGGGGCT |
| PML-intron-QP | TCCCAACTCTGGACTGCTCT | ATAGGCTAGGCGCATGCAAA |
| PML-RARA-FL | ATGGAGCCTGCACCCGCCCGATCT | TCACGGGGAGTGGGTGGCCGGGCTGC |
| METTL3-FL | AATTAAGCTTATGGATTACAAGGATGACGATGACAAGTCGGACACGTGGAGCT (Hind III) | AATTTCTAGACTATAAATTCTTAGGTTTAGAGATGATAC (Xba I) |
| METTL14-FL | AATTGGATCCATGGATTACAAGGATGACGATGACAAGGATAGCCGCTTGCAGGA (BamH I) | AATTCTCGAGTTATCGAGGTGGAAAGCCAC (Xho I) |
| WTAP-FL | AATTGGATCCATGGATTACAAGGATGACGATGACAAGACCAACGAAGAACCTCTTCC (BamH I) | AATTCTCGAGTTACAAAACTGAACCCTGTACATTTACA (Xho I) |
| SRSF3-FL | GCTAGCATGCATCGTGATTCCTGTCCA (Nhe I) | GGATCCCTATTTCCTTTCATTTGACCTA(BamH I) |
| AML1-ETO | Plasmid #12433 from addgene | |
| MLL-ENL | Plasmid #20873 from addgene | |
| MLL-AF9 | Plasmid #71443 from addgene | |
| RARA-FISH | 5‘FITC+GACACGUUGUUCUGAGCUGUUGUUCGUAGUGUAUUUGC-3’ | |
| PML-FISH | Specific RNA FISH from Ribobiotech. company | |

**Supplementary Table S2. siRNA or shRNA sequences for target genes.**

| Genes | sense | antisense |
| --- | --- | --- |
| ShMALAT1 | GAUCAGGAUUUGAGCGGAA | UUCCGCUCAAAUCCUGAUC |
| SiMALAT1-1 | GAUCAGGAUUUGAGCGGAA | UUCCGCUCAAAUCCUGAUC |
| SiMALAT1-2 | AAGAUAUUGCUUAGCGUUA | UAACGCUAAGCAAUAUCUU |
| SiMETTL3-1 | CUGCAAGUAUGUUCACUAUGA | UCAUAGUGAACAUACUUGCAG |
| SiMETTL3-2 | AGGAGCCAGCCAAGAAAUCAA | UUGAUUUCUUGGCUGGCUCCU |
| SiMETTL14-1 | AAGGAUGAGUUAAUAGCUAAA | UUUAGCUAUUAACUCAUCCUU |
| SiMETTL14-2 | UGGUGCCGUGUUAAAUAGCAA | UUGCUAUUUAACACGGCACCA |
| SiWTAP-1 | AAGCUUUGGAGGGCAAGUACA | UGUACUUGCCCUCCAAAGCUU |
| SiWTAP-2 | AAGGUUCGAUUGAGUGAAACA | UGUUUCACUCAAUCGAACCUU |
| SiWTAP-3 | GGGCAAGUACACAGAUCUUAA | UUAAGAUCUGUGUACUUGCCC |
| SiYTHDC1-1 | CGACCAGAAGAUUAUGAUA | UAUCAUAAUCUUCUGGUCG |
| SiYTHDC1-2 | CGAGUAUGCAAAUAUUGAA | UUCAAUAUUUGCAUACUCG |
| siDDX39B-1: | AAGGGCUUGGCUAUCACAUUU | AAAUGUGAUAGCCAAGCCCUU |
| SiDDX39B-2: | GUCACACUCGGGAGUUGGC | GCCAACUCCCGAGUGUGAC |
| SiThoc5-1 | CCAAAUGUUUGGAGUUUAA | UUAAACUCCAAACAUUUGG |
| SiThoc5-2 | GAAAGUAGAUGCCUAUCAU | AUGAUAGGCAUCUACUUUC |
| SiALYREF-1 | GGAACUCUUUGCUGAAUUU | AAAUUCAGCAAAGAGUUCC |
| SiALYREF-2 | GCACGAUCUUUUCGACAGU | ACUGUCGAAAAGAUCGUGC |
| siThoc1-1 | GCCAUUGAACAGGCAGACC | GGUCUGCCUGUUCAAUGGC |
| siThoc1-2 | CACAUCCUGUUGCAGUAUC | GAUACUGCAACAGGAUGUG |
| siTho2-1 | ACACUGGAAUCAUUAGGGC | GCCCUAAUGAUUCCAGUGU |
| siTho2-2 | GCCAGUCGAUACGGAAGGU | ACCUUCCGUAUCGACUGGC |
| siSRSF3- 1 | CCUGUCCAUUGGACUGUAA | UUACAGUCCAAUGGACAGG |
| siSRSF3- 2 | GCUAGAUGGAAGAACACUA | UAGUGUUCUUCCAUCUAGC |
| siSRSF7 -1 | GGUACGGAGGAGAAACCAA | UUGGUUUCUCCUCCGUACC |
| siSRSF7- 2 | GCAUCUCCUCGACGAUCAA | UUGAUCGUCGAGGAGAUGC |
| siCPSF6-1 | CGAAGAGUUCAACCAGGAA | UUCCUGGUUGAACUCUUCG |
| siCPSF6 -2 | CAUAGUAGAUCACGAGAAA | UUUCUCGUGAUCUACUAUG |
| siCHTOP-1 | GACAACCAAUUGGAUGCAUAU | AUAUGCAUCCAAUUGGUUGUC |
| siCHTOP-2 | CAGACAGAUCCCGAAACCAAUGAUU | AAUCAUUGGUUUCGGGAUCUGUCUG |
| siRBM15-1 | GGAAGAAAGCUAAUCUGUUUAGUAU | AUACUAAACAGAUUAGCUUUCUUCC |
| siRBM15-2 | GCAGCGGAAAGACCGAUAGCGGCGG | CCGCCGCUAUCGGUCUUUCCGCUGC |
| siGANP-1/MCM3AP | GAGAGGACCUAAGUCAAUA | UAUUGACUUAGGUCCUCUC |
| siENY2-1 | GGCACACUGUAAAGAGGUA | UACCUCUUUACAGUGUGCC |
| siENY2-2 | AGAGAACGCCUCAAAGAGU | ACUCUUUGAGGCGUUCUCU |
| siTPR-1 | GGCAUACACUUACUAGAAA | UUUCUAGUAAGUGUAUGCC |
| siTPR-2 | UCAAGGAGGUUUAGGAAUG | CAUUCCUAAACCUCCUUGA |

**Supplementary Table S3. Antibodies used in WB and IF.**

| Antibody | Company | Catalog |
| --- | --- | --- |
| anti-METTL3 antibody | Proteintech | 15073-1-AP |
| anti-METTL14 antibody | Proteintech | 26158-1-AP |
| anti-WTAP antibody | Proteintech | 60188-1-lg |
| anti-Flag (mouse) | Sigma | A8592 |
| polyclonal rabbit anti GAPDH) antibody | Sigma-Aldrich | G9545 |
| anti-Flag (rabbit) antibody | Proteintech | 20543-1-AP |
| anti-RARA antibody | Santa cruz | sc-550 |
| anti-PML antibody | Santa cruz | sc-966 |
| anti-HA (mouse) antibody | Sigma | H3663 |
| anti-HA (rabbit) antibody | CST | C29F4 |
| anti-MLL antibody | Bethyl | A300-086A-3 |
| anti-SC35 antibody | Abcam | Ab11826 |
| anti-SF2 antibody | Santacruz | Sc-33652 |
| anti-mouse HRP-conjugated secondary antibody | Sigma Aldrich | A3682 |
| anti-rabbit HRP-conjugated secondary antibody | Sigma Aldrich | A9542 |
| anti-CD11b-APC | BD | 550019 |
| anti-CD14-APC | BD | 555399 |
| anti-m6A | Abcam | Ab151230 |
| AlexaFluor 594-conjugated secondary antibodies(mouse) | Invitrogen | A11005 |
| AlexaFluor 594-conjugated secondary antibodies(rabbit) | Invitrogen | A21207 |
| AlexaFluor 488-conjugated secondary antibodies(mouse) | Invitrogen | A11001 |

**Supplementary Table S4. Adaptor proteins regulating mRNA export.**

| **m6A binding peptides** | **Adaptors** | **Protein Family** |
| --- | --- | --- |
| 9 | hHpr1/THOC1 | TREX |
| 5 | Thoc2 |  |
| 7 | Thoc7 |  |
| 6 | Thoc5 |  |
| 0 | Thoc6 |  |
| 0 | hTex1/THOC3 |  |
| 4 | Uap56/DDX39B |  |
| 13 | AlyREF/THOC4 |  |
| 17 | 9G8/SRSF7 | SR |
| 18 | SRp20/SRSF3 |  |
| 5 | CFIm68/CPSF6 |  |
| 7 | CHTOP |  |
| 21 | RBM15 |  |
| 4 | GANP | TREX-2 |
| 0 | PCID2 |  |
| 0 | DSS1 |  |
| 6 | ENY2 |  |
| 0 | CBP80 |  |
| 0 | CRM1 |  |

**Supplementary Table S5. The predicted m6A motif located at PML-RARα mRNA**

| **M6A methylation** | **conserved binding sites** | **motif of PML fusion exon 4 up and down 1kb** | **motif of PML fusion Exon 4** | **motif of PML fusion exon 5 up 1kb** | **motif of PML fusion Exon 5** | **motif of PML fusion exon 6 up and down 1kb** | **motif of fusion PML Exon 6** | **motif of RARA fusion exon 3 up and down 1kb** | **motif of RARA fusion exon 3** |
| --- | --- | --- | --- | --- | --- | --- | --- | --- | --- |
| METTL14 | GGACT | 4 | 0 | 2 | 0 | 1 | 0 | 2 | 0 |
|  | GGACC | 2 | 1 | 1 | 0 | 1 | 1 | 0 | 0 |
|  | GGACA | 4 | 0 | 1 | 0 | 4 | 0 | 4 | 1 |
| METTL3 | GAGGAC | 0 | 0 | 1 | 0 | 0 | 0 | 0 | 0 |
|  | GTGGAC | 0 | 0 | 0 | 0 | 2 | 0 | 0 | 0 |
|  | GCGGAC | 0 | 0 | 0 | 0 | 0 | 0 | 0 | 0 |
|  | CAGGAC | 1 | 0 | 0 | 0 | 0 | 0 | 1 | 1 |
|  | CTGGAC | 5 | 0 | 1 | 0 | 1 | 0 | 0 | 0 |
| WTAP | GACTCT | 1 | 0 | 0 | 0 | 0 | 0 | 2 | 0 |
|  | GGCTCT | 1 | 0 | 0 | 0 | 2 | 0 | 2 | 0 |
|  | GACTGT | 1 | 0 | 0 | 0 | 0 | 0 | 0 | 0 |
| **Total** |  | **19** | **1** | **6** | **0** | **11** | **1** | **11** | **2** |
