# Supplementary material for: Nuclear export of chimeric mRNAs depends on an lncRNA-triggered autoregulatory loop in blood malignancies
Source: Cell Death Dis. 2020 Jul 23;11(7):566. doi: 10.1038/s41419-020-02795-1 (PMC7378249; doi:10.1038/s41419-020-02795-1)
Supplement: Supplementary file 2 — Supplementary Figure Legends [file 41419_2020_2795_MOESM2_ESM.docx]

**Supplementary Figure Legends**

**Supplementary Fig. S1.** **Over-expressed PML-RARA regulates cellular differentiation.**  **a** Immunoprecipitation experiments were carried out in NB4 cells lentivirally infected with a Flag-tagged PML-RARA PCDH construct, and the subsequent Western blot assay showed that the specific PML-RARA was observed. **b** The same results for Western blot using antibodies for HA or RARA showed that compared with the negative control, HA-tagged PML-RARA was also overexpressed in liposome-transfected HEK-293T cells. **c** Schematic showing MALAT1 sections in MS2-Trap assays. qRT-PCR results showed that MALAT1 was much more enriched in 12×MS2bs-MALAT1 samples than in negative controls 12×MS2bs-NC after the immunoprecipitation of Flag-tagged MS2. Data are shown as the means ± s.e.m.; n = 3 independent experiments.

**Supplementary Fig. S2. Reduction of MALAT1 promoted cellular differentiation by inhibiting the mRNA export process of fusion proteins. a** qRT-PCR measurement of MALAT1 levels after suppressing MALAT1 by two siRNAs with different primers of MALAT1 in NB4 cells. Data are shown as the mean ± s.e.m.; n = 3 independent experiments. **b** Immunofluorescence analysis of PML-RARA in NB4 cells and representative images showing that the dots indicating PML-RARA were markedly decreased after knockdown of MALAT1 compared with those in controls. Scale bar represents 4 μm. **c** qRT-PCR delta CT analysis of PML-RARA mRNA levels in the cytoplasm versus the Nucleus. Data are shown as the means ± s.e.m.; n = 3 independent experiments. **d** qRT-PCR analysis of MALAT1 levels in the siNC, siMALAT1, and combination of siMALAT1 and MALAT1 3’ end section (oeMALAT1) samples of NB4 cells. Data are shown as the mean ± s.e.m.; n = 3 independent experiments. **e** PML-RARα mRNA detection by qRT-PCR after separating the nuclear and cytoplasmic RNAs in the siNC, siMALAT1, and combination of siMALAT1 and oeMALAT1 transfected NB4 cells. **f, g** Representative graphs for the flow cytometric analysis of myeloid markers of CD11b and CD14 in molm13, a monocyte cell line; and CD11b, CD14, and CD15 in NB4, a promyelocyte cell line. Histogram plots show the statistical values. Error bars reflect ± SEM in three independent experiments. **h, i** Wright-Giemsa staining of molm13 and HL60 cells after transduction with MALAT1 siRNAs or control. Scale bar, 20 μm. **j** Cellular apoptosis measurement by flow cytometry. Data are shown as the means ± s.e.m.; n = 3 independent experiments.

**Supplementary Fig. S3.** **Screening of mRNA export regulatory proteins required for fusion gene transport from the nucleus to the cytoplasm.** **a-c** Measuring the effects of knocking down mRNA export-related proteins by siRNAs using qRT-PCR results normalized to the reference gene GAPDH. Data are shown as the means ± s.e.m.; n = 3 independent experiments. **d** qRT-PCR analysis of SRSF3 mRNA levels in the siNC, siMALAT1, and combination of siMALAT1 and overexpression SRSF3 (oeSRSF3) transfected NB4 cells. Data are shown as the means ± s.e.m.; n = 3 independent experiments. **e** PML-RARα mRNA detection by qRT-PCR after separating the nuclear and cytoplasmic RNAs in the siNC, siMALAT1, and combination of siMALAT1 and overexpression SRSF3 (oeSRSF3) transfected NB4 cells.

**Supplementary Fig. S4.** **MALAT1-regulated m6A levels are responsible for fusion gene export and cellular differentiation.** **a** Measuring the effect of knocking down m6A methyltransferases by siRNAs using qRT-PCR normalized to the reference gene GAPDH. Data are shown as the means ± s.e.m.; n = 3 independent experiments. **b** Sequencing results of MeRIP-PCR for PML exon 4 and exon 6. **c** The qRT-PCR delta CT analysis of PML-RARA mRNA levels in the cytoplasm versus the nucleus in NB4 cells after decreasing m6A methyltransferase levels. Data are shown as the means ± s.e.m.; n = 3 independent experiments. **d, e** PML-RARA and MLL-AF9 mRNA detection by qRT-PCR after inhibiting m6A levels or YTHDC1 in NB4 cells and moml13 cells, respectively. **f** The protein levels of MLL-AF9 were monitored via Western blot after knocking down m6A methyltransferases and YTHDC1 by siRNAs. Three independent experiments were carried out. **g** Wright-Giemsa staining showed that cellular differentiation was significantly promoted after reducing m6A levels in NB4 cells. The nuclear shape of these cells was altered from a round to a lobulated shape. The experiments were performed independently at least three times. Scale bar represents 20 μm. **h** Representative graphs for the flow cytometric analysis of myeloid markers of CD11b, CD14, and CD15 in NB4, a promyelocyte cell line, when the METTL3, METTL14, and WTAP were knocked down. Histogram plots show the statistical values. Error bars reflect ± SEM in three independent experiments.

**Supplementary Fig. S5. METTL3, METTL14, and WTAP regulated cellular differentiation.** Representative graphs for the flow cytometric analysis of myeloid markers of CD11b, CD14, and CD15 in MALAT1 overexpressing NB4 cells that silenced the METTL-M proteins. Histogram plots show the statistical values. Error bars reflect ± SEM in three independent experiments (***, p<0.001).

**Supplementary Fig. S6.** **m6A methyltransferase interacts to PML-RARA.** Western blot analysis for PML-RARA following the immunoprecipitation of specific m6A methyltransferases in the lentiviral-infected NB4 cells. Flow through (FT1) and flow through after washing three times (FT3) were loaded as controls.

**Fig. S7. MALAT1 functions in cellular differentiation via m6A during malignant hematopoiesis in vivo. a** Determining the effect of knocking down MALAT1 in NB4 cells infected by lentiviral constructs carrying shRNA-targeted MALAT1 using qRT-PCR normalized to GAPDH. Data are shown as the means ± s.e.m.; n = 3 independent experiments. **b** Representative images of NOD-SCID mice with ascites fluid injected by human NB4 cells that were suppressed for MALAT1 or enhanced m6A methyltransferases. **c** Testing the effects of knocking down MALAT1 of ascites fluid of NOD-SCID mice infected by lentiviral constructs carrying shRNA targeting MALAT1 using the methods of qRT-PCR normalized to reference gene GAPDH. Data are shown as the means ± s.e.m.; n = 3 independent experiments. **(d, e)** Wright-Giemsa assays were carried out to verify the karyotypes of murine ascites fluid cells after knocking down MALAT1 or overexpressing METTL3, 14 or WTAP in human NB4 cells. Scale bar represents 10 μm. **f** Differentiation marker CD14 of NOD-SCID murine ascites fluid was analyzed by flow cytometry after altering MALAT1 expression or m6A levels. Data are shown as the mean ± s.e.m.; n = 3 independent experiments. **g** In the tail vein injection mouse model, bone marrow cells were used to detect PML-RARA protein levels by Western blot after suppressing MALAT1 expression (shMALAT1-BM#1,2,3).
