# Supplementary figures and images for: Nuclear export of chimeric mRNAs depends on an lncRNA-triggered autoregulatory loop in blood malignancies
Source: Cell Death Dis. 2020 Jul 23;11(7):566. doi: 10.1038/s41419-020-02795-1 (PMC7378249; doi:10.1038/s41419-020-02795-1)

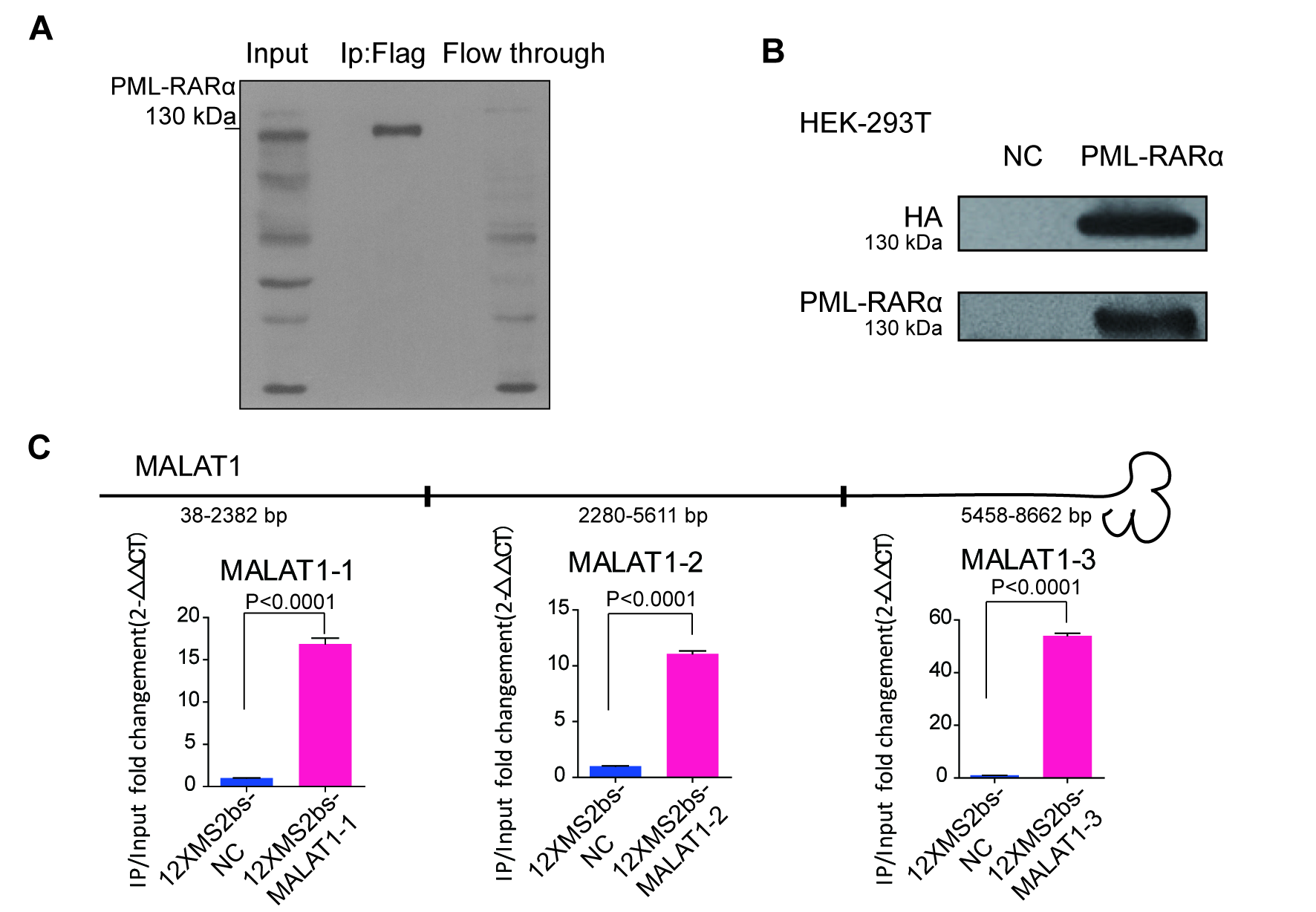

Supplement: Supplementary file 3 — Figure S1 [file 41419_2020_2795_MOESM3_ESM.tif]

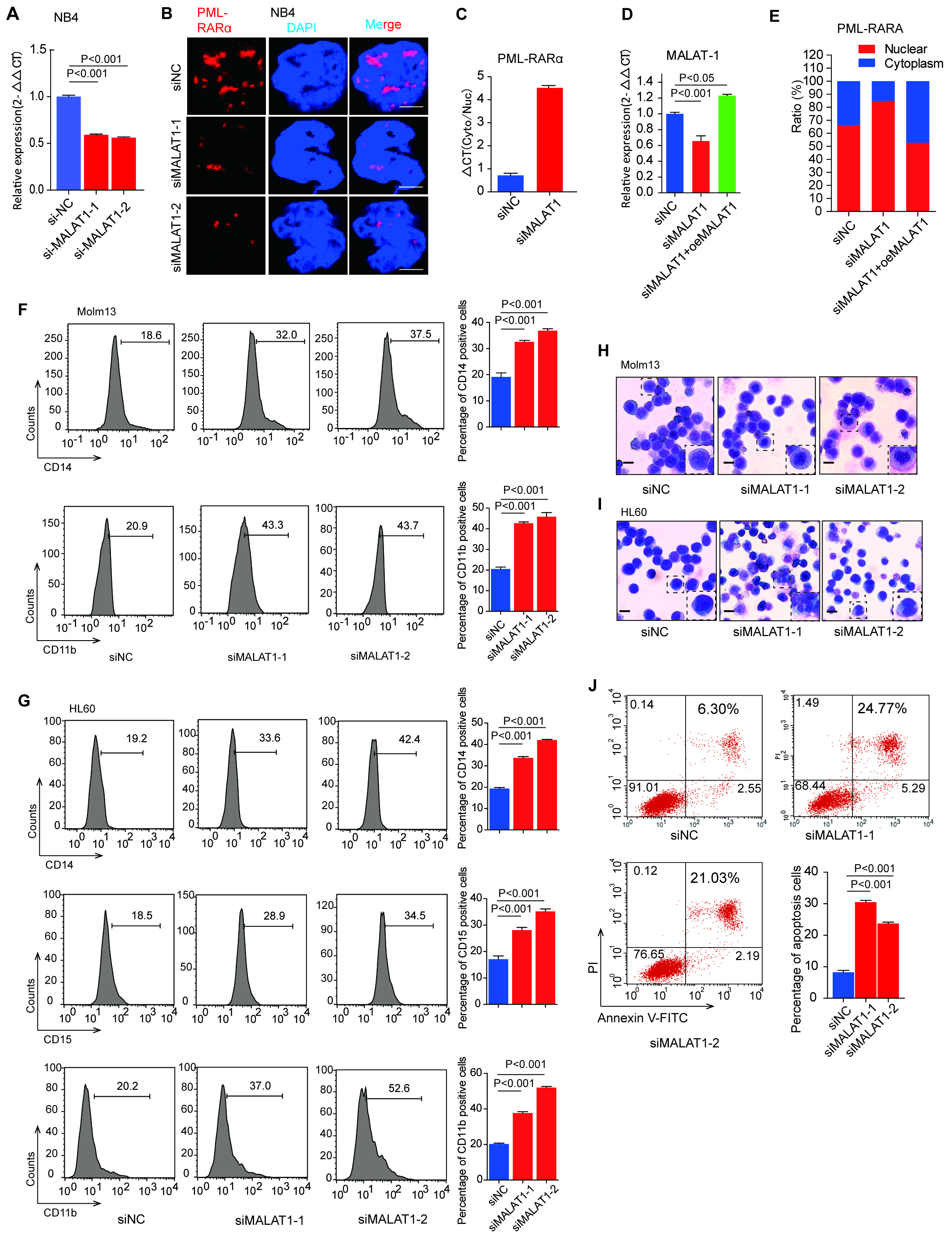

Supplement: Supplementary file 4 — Figure S2 [file 41419_2020_2795_MOESM4_ESM.tif]

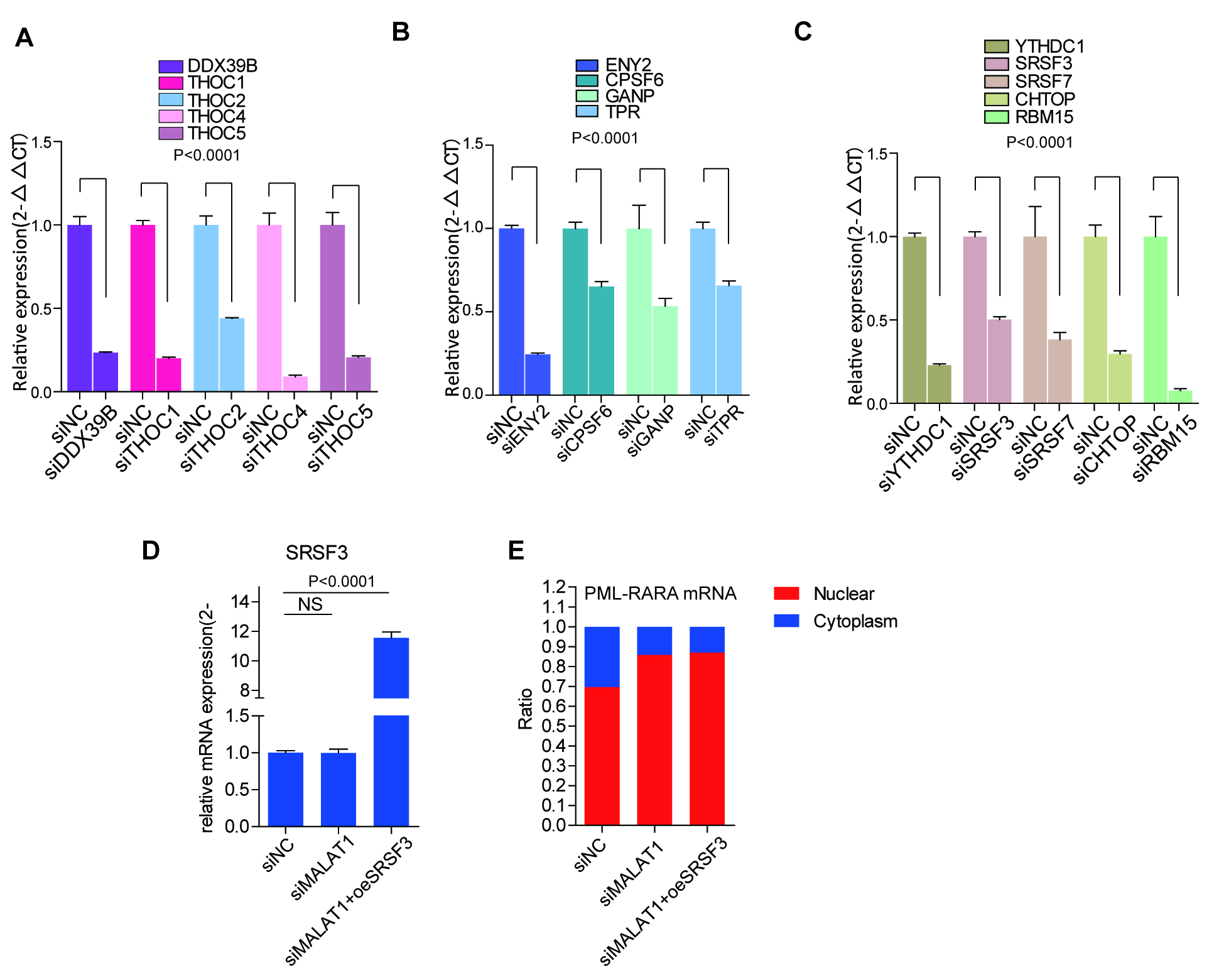

Supplement: Supplementary file 5 — Figure S3 [file 41419_2020_2795_MOESM5_ESM.tif]

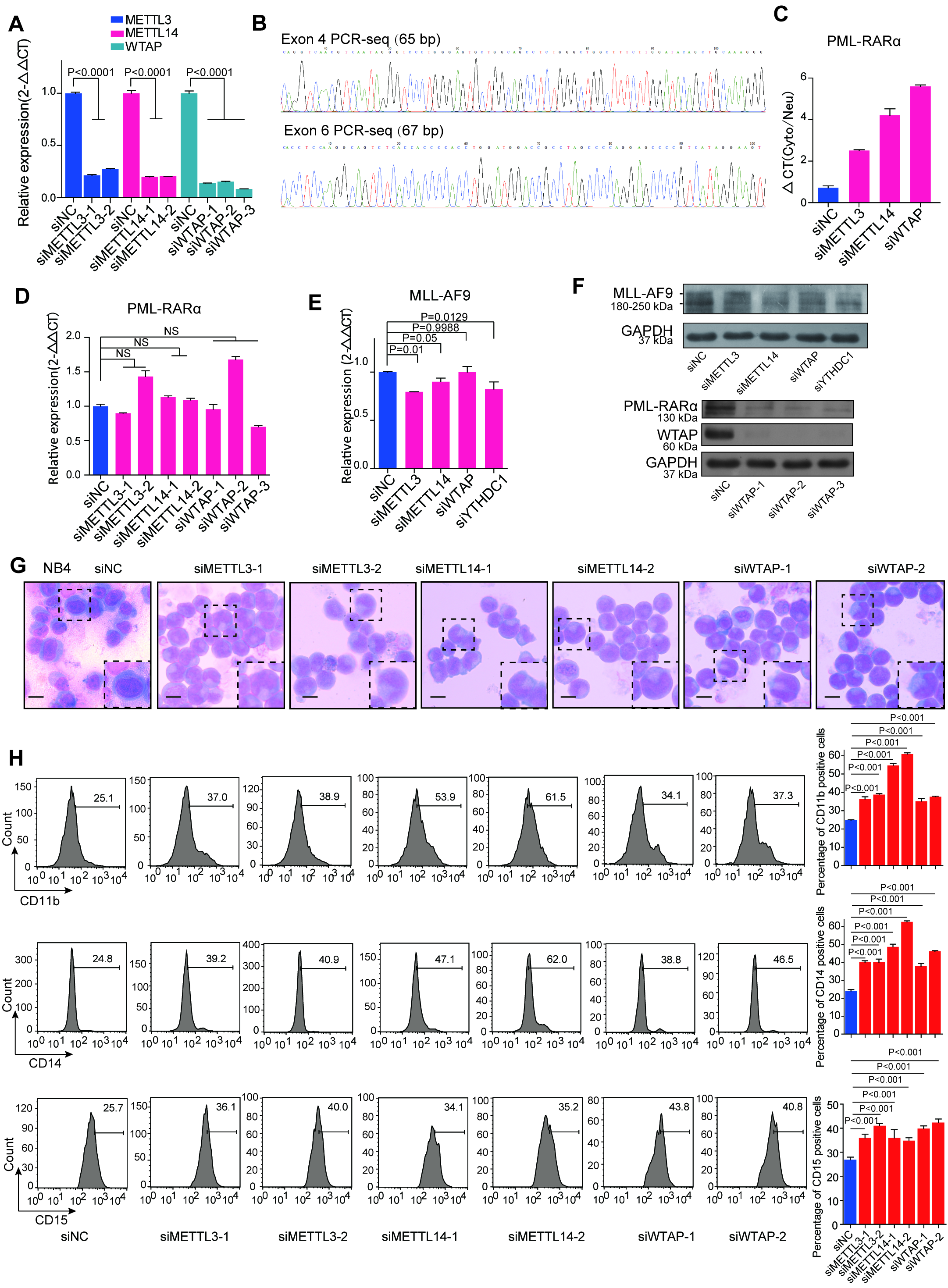

Supplement: Supplementary file 6 — Figure S4 [file 41419_2020_2795_MOESM6_ESM.tif]

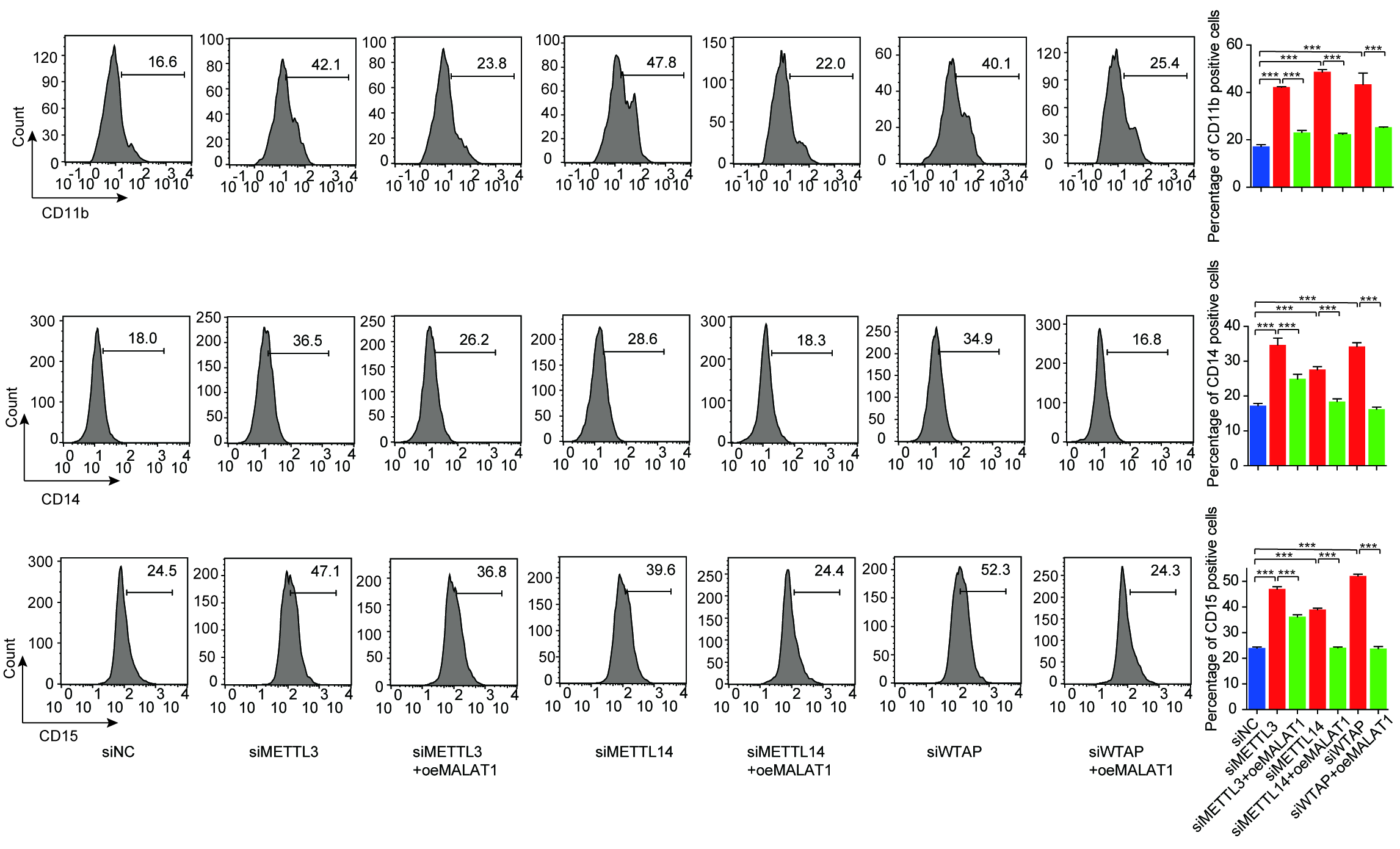

Supplement: Supplementary file 7 — Figure S5 [file 41419_2020_2795_MOESM7_ESM.tif]

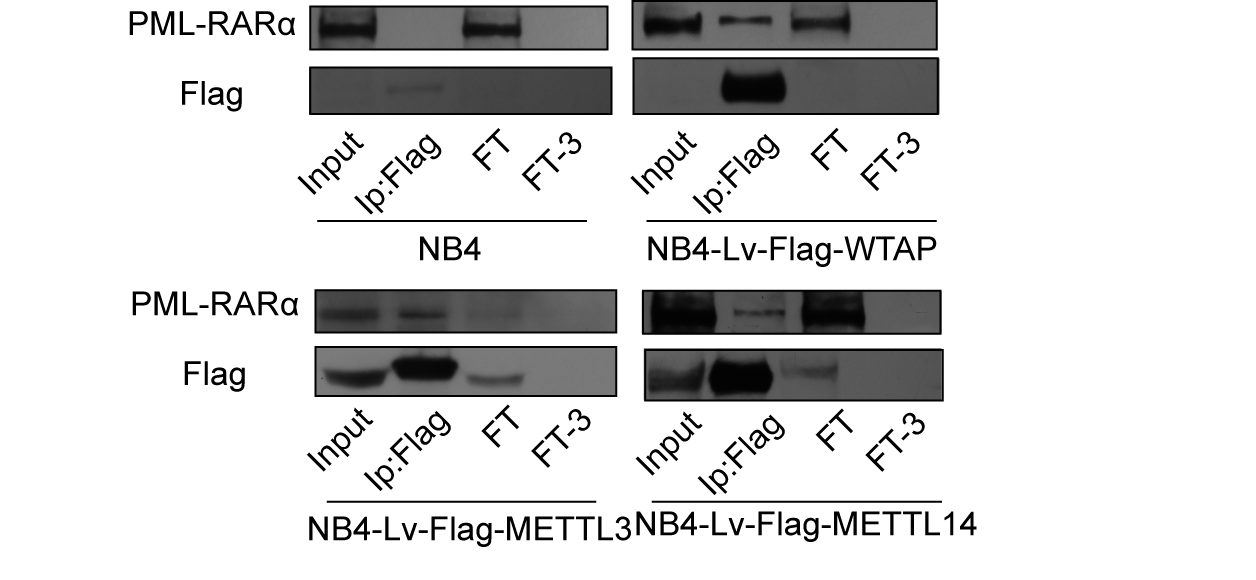

Supplement: Supplementary file 8 — Figure S6 [file 41419_2020_2795_MOESM8_ESM.tif]

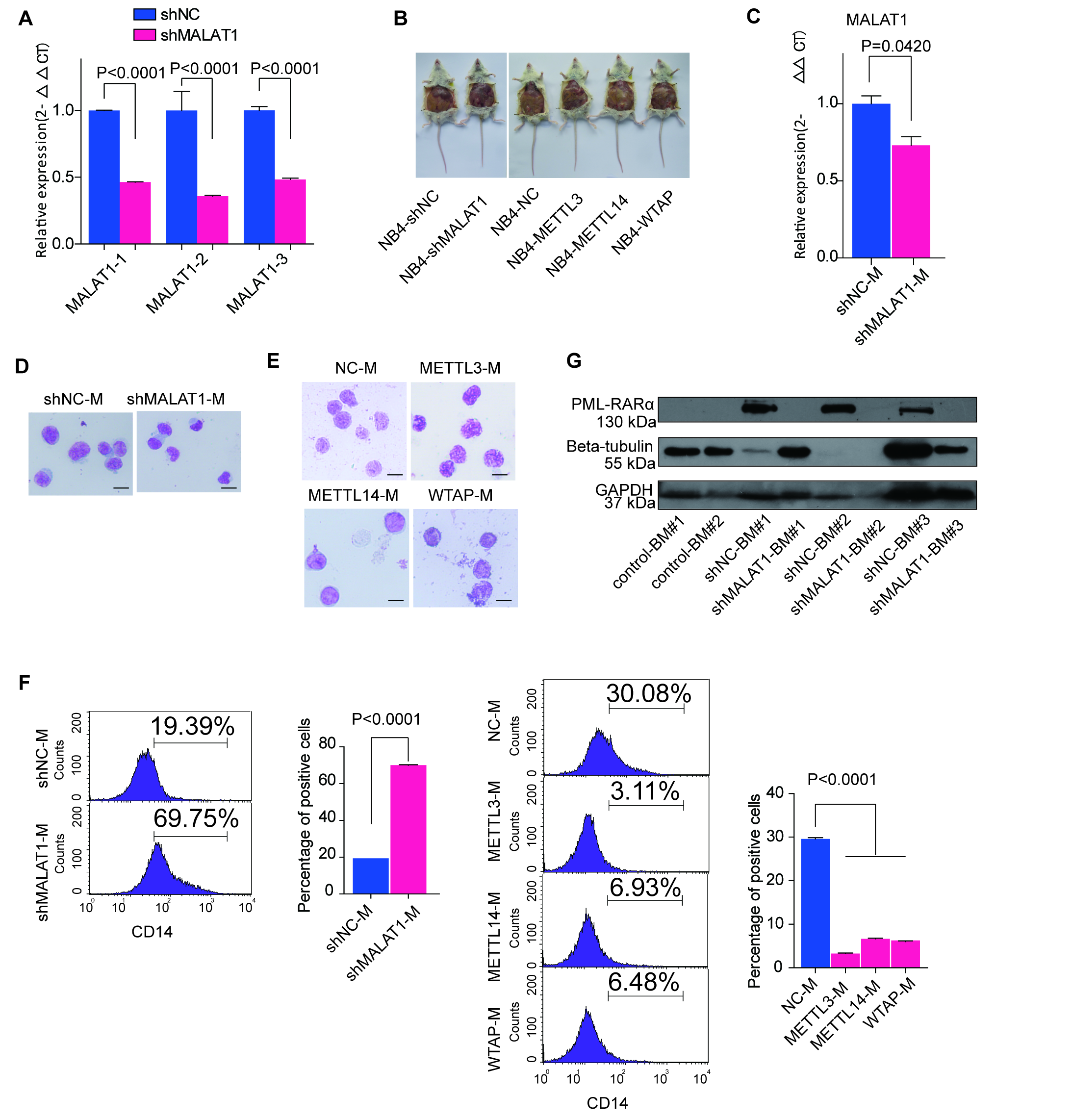

Supplement: Supplementary file 9 — Figure S7 [file 41419_2020_2795_MOESM9_ESM.tif]
